# eDNA as a tool for non-invasive monitoring of the fauna of a turbid, well-mixed system, the Elbe estuary in Germany

**DOI:** 10.1371/journal.pone.0250452

**Published:** 2021-04-16

**Authors:** Martin Schwentner, Reza Zahiri, Satoshi Yamamoto, Martin Husemann, Björn Kullmann, Ralf Thiel

**Affiliations:** 1 Center of Natural History, Universität Hamburg, Hamburg, Germany; 2 Naturhistorisches Museum Wien, Vienna, Austria; 3 Entomology Diagnostic Laboratory, Ottawa Plant Laboratory, Canadian Food Inspection Agency, Ottawa, Ontario, Canada; 4 Graduate School of Science, Kyoto University, Kyoto, Japan; CSIR-National Institute of Oceanography, INDIA

## Abstract

The Elbe is one of the longest European rivers and features a large, turbid and well-mixed estuary, which runs through the inner city of Hamburg. The Elbe has been closely monitored using classical catch techniques in the past. Here we tested a COI-based eDNA approach for assessing the biodiversity within the Elbe. We sampled three stations in the Elbe, included low and high tide events, as well as two adjoining lakes to compare the recovered faunas. To analyze the data, we employed two different pipelines: the automated mBRAVE pipeline utilizing the BOLD database and one including NCBI BLAST. The number of OTUs with species or higher-level identifications were similar between both approaches with 352 OTUs and 355 OTUs for BLAST and mBRAVE, respectively, however, BLAST searches recovered another 942 unidentified metazoan OTUs. Many taxa were well represented; however, fish species were poorly represented, especially in the Elbe estuary samples. This could be a result of the universal COI primers, which also yielded high read numbers for non-metazoan OTUs, and small-bodies taxa like Rotifera, which might have been sampled together with the eDNA. Our results show a strong tidal influence on the recovered taxa. During low tide, downstream stations resembled sites further upstream, but the former showed a very different OTU composition during high tide and early tide. Such differences might be due to varying impacts of upstream-originating eDNA during tide cycles. Such factors need to be considered when routinely employing eDNA for monitoring programs.

## Introduction

The use of DNA sequence data is becoming increasingly important for species diagnoses and documenting and monitoring global biodiversity [1]. The mitochondrial cytochrome *c* oxidase subunit I (COI) gene region has proved particularly useful in delimiting and discriminating animal species in taxonomic, ecological and evolutionary studies [2, 3] and is routinely used as a standardized DNA barcode [2, 4, 5]. Public DNA repositories like GenBank or BOLD (Barcode of Life Database) now contain such DNA barcodes of a large number of species, allowing the automated identification of many taxa. This becomes particularly attractive in combination with high-throughput sequencing (HTS) technologies, as here thousands or even millions of sequences can be generated simultaneously. One of the main advantages of DNA barcoding is the ability to identify specimens irrespective of their morphological appearance, once a DNA barcode is established for a species. This enables the identification of morphologically cryptic or sexually dimorphic species, as well as juveniles, larvae or even small fragments or excretions. Even environmental DNA in water samples can be used to identify species living in a certain habitat.

High-throughput sequencing of genetic material applied to environmental DNA (eDNA), such as DNA extracted from water samples, provides a non-invasive method to rapidly identify multiple taxa living in the particular habitat. eDNA surveys focus on the presence or absence of genetic material of target species within a sampled area. Such eDNA surveys not only replace time consuming and often difficult morphological identifications, but even eliminate the need of physically sampling of individuals [6–10]. Therefore, they are especially useful for detecting rare, elusive, threatened or emerging invasive species [9, 11–13]. Further, this method promises great advantages in habitats, which are difficult to sample with classic methods, e.g. in large aquatic systems, such as large lakes, or rivers [14–17]. Using eDNA eliminates the need to employ various sampling techniques (like dip or hand nets, seine nets, electrofishing, etc.) to cover all taxa and species of different size, habitat or life-style. Moreover, the rapid nature of eDNA sample collection and processing, and the continually decreasing cost of sequencing [18] makes this approach suitable for increasing spatial and temporal coverage of bio-surveillance programs.

However, in rivers and their estuaries, eDNA approaches encounter some limitations and specific problems due to the flow of water and turbidity [19]. It was for example shown that transportation of eDNA is species-specific and recovery at different distances from the source of the DNA may be strongly taxon dependent [20]. Further, it should be clearly stated that non-detection of a species does not confirm its absence; nor does detection confirm its presence at that time at the specific location [19]. In flowing water masses, detection of eDNA of a species only confirms that the species was present upstream from the point of sampling at some time. Finally, there is little knowledge so far on the specificity and recovery rates for invertebrates in river systems [19]. While these factors may at least partially limit the power of eDNA analyses in flowing water bodies, these studies also show that it is worth investing in this technique further to better understand the results and solve these problems for future projects. Hence, we here employ eDNA analyses in a large estuary in Northern Germany–the Elbe estuary–to assess its power for future biodiversity surveys.

The Elbe estuary has been intensively studied and monitored since more than 60 years. Some of the earliest reliable water chemistry data date back to the 1950s [21]. Great effort has been invested into the monitoring of especially the fish fauna (e.g. BMBF-project Klimzug Nord, [22, 23]). As a result of different monitoring projects, it is known that about 80 fish species occur in the Elbe estuary, which is thus considered one of the most diverse European estuaries in terms of fish species [22, 24]. However, in such an open and turbid aquatic system with a strong tidal influence and permanently changing physical and chemical characteristics, biomonitoring requires a high spatial and temporal resolution and is therefore a very costly and time-consuming task, especially when using traditional sampling techniques and when studying invertebrates as well. Further, many of the classic monitoring methods result in the unnecessary death of specimens. In this regard, biomonitoring based on eDNA would provide a viable alternative as it is non-lethal, and once standardized much less time consuming, while potentially covering all taxa and not only specifically selected groups.

The objective of this project was to study the power of eDNA as a tool for biodiversity assessment in a tidal, well-mixed estuary system—the lower Elbe River—focusing on fish and invertebrate species. We sampled stations in the Elbe estuary with strong and somewhat weaker tidal influence and included low as well as high tide events. Two nearby lakes were studied as well to compare recovery success of species and to assess the overlap between riverine and lake species. We further compared two different approaches, a BLAST based pipeline and mBRAVE using BOLD, regarding the recovery of diversity for various taxonomic groups.

## Materials and methods

### Study area

The Elbe is one of the longest European rivers having today a total length of 1,094 km and a catchment area of 148,268 km^2^, the fourth largest river basin in Central Europe [25]. The 140 km long and tidally influenced estuary of the Elbe is the largest estuary of the German Bight of the southern North Sea. The Elbe estuary is heavily influenced by tides and is classified as a turbid, well-mixed, mesotidal/macrotidal estuarine system [26, 27]. The tidal part of the Elbe is an important waterway having an inland delta about 20–40 km downstream of the weir at the city of Geesthacht. The inland delta includes the Port of Hamburg, Germany’s largest seaport. The range of the semi diurnal tide at the Hamburg Harbor is 3.6 m. High tidal current velocities (up to 1.8 m s^-1^) [28] cause a steep horizontal salinity gradient. Permanently changing physical and chemical characteristics make the Elbe estuary a very challenging environment for animal life. In addition to natural changes, the Elbe estuary has been subjected to man-made modifications for centuries by for instance diking, land reclamation, realignment, riverine water quantity management, pollution and navigation channel deepening [25]. In the last decades, especially the deepening of the Elbe estuary for commercial shipping has contributed to a reduction of the surface/volume ratio, a decreasing dissolved oxygen level and an increasing turbidity.

### Sampling

**Fig 1:**
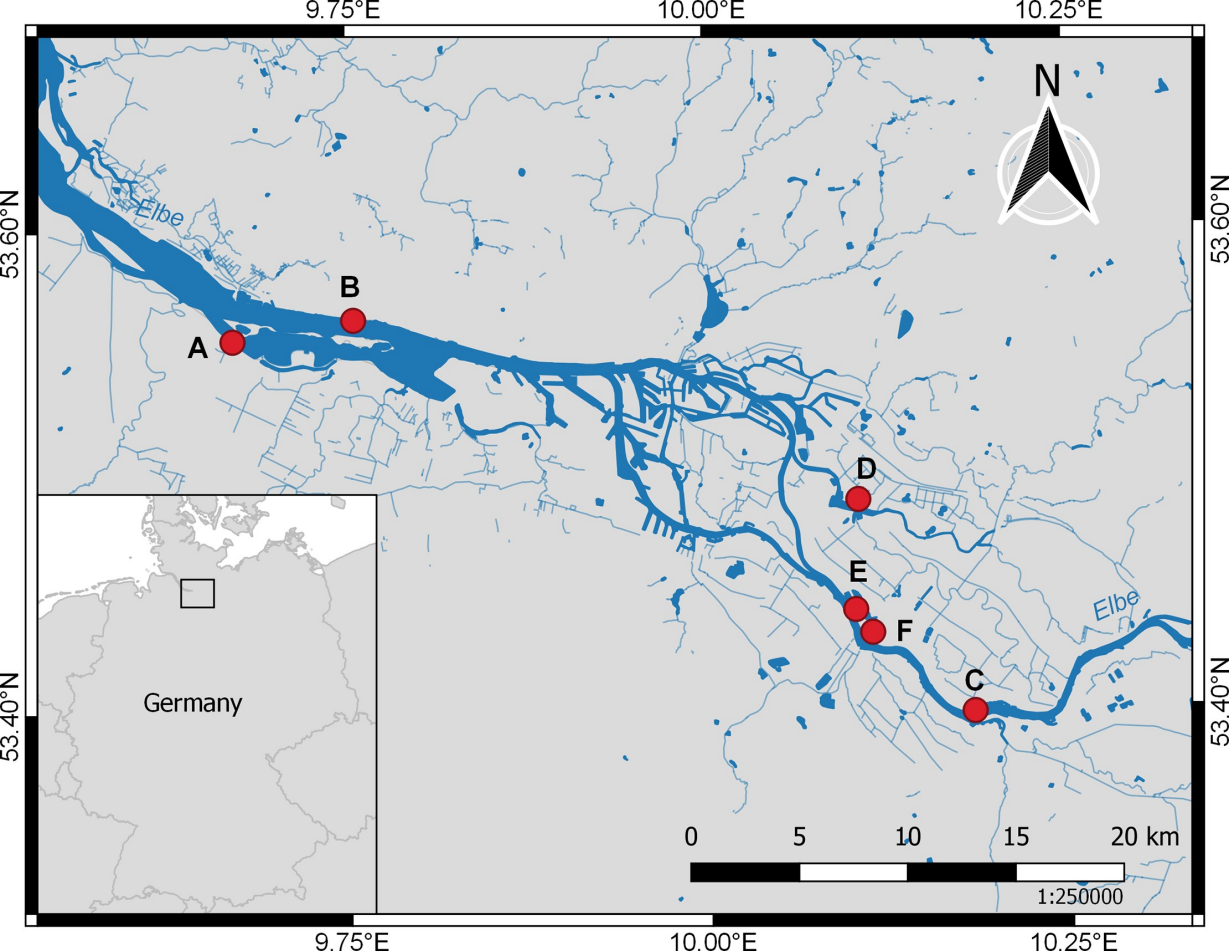
eDNA sampling localities along the Elbe estuary and adjacent lakes. For details on sampling events see S1 Table. The map was created using the Free and Open Source QGIS (Version 3.10 A Coruña; https://www.qgis.org/de/site/). It contains information from OpenStreetMap and OpenStreetMap Foundation, which is made available under the Open Database License. Stations A-C Elbe estuary, Station D Lake Eichbaum, Stations E and F Lake Hohendeich.

Sampling of eDNA was conducted at six stations belonging or close to the territory of the city of Hamburg, Germany. Three stations were sampled in the tidal freshwater part of the Elbe estuary downstream (stations A, B) and upstream (station C) of the Hamburg harbor. Three further stations were sampled in two lakes near the estuarine section of the Elbe upstream of the Hamburg harbour: Lake Eichbaum (station D) and Lake Hohendeich (stations E, F; Fig 1; S1 Table). Both lakes are not connected to the Elbe estuary. As all water samples were taken outside of protected areas, no permits were required.

At each station, water was collected with a bucket. The bucket was cleaned with commercial bleach-solution at each station and washed twice in the location’s water. Water was filtered via a sterile 50 ml syringe through a sterile Sterivex-VF filter (0.45 μm cartridge filter, PVDF membrane, 10 cm^2^ filter area; Millipore, Burlington, Massachusetts, USA). At each station, three replicates were taken, filtering 200 ml water for each replicate. For each replicate a fresh water sample was collected. 200 ml was the maximum amount of water that could be pressed through the filter at Elbe estuary stations due to the high load of particles. For consistency, we filtered the same amount of water also at the lake stations, though here filters clogged slower. To assess if higher species diversities would have been recovered if more water had been filtered, we took a fourth replicate at each lake station filtering 1000 ml (this water was taken from the same water as one of the other replicates). The filters were conserved with RNAlater, sealed, placed into a separate plastic zip-lock bag and put on ice immediately. At two stations (Stations B and C), sampling was conducted twice, once at high and once at low tide. These sampling events occurred at most 1.5h from the respective high or low tide event (S1 Table). Two negative controls were taken in the field using distilled water, following the same procedure as for the samples (e.g., taking the distilled water from the cleaned bucket). These were taken after sampling Lake Hohendeich (Station E).

### DNA extraction, PCR amplification and sequencing

RNAlater was removed from the Sterivex filter via centrifugation for 1 min at 6000 × *g*. For this, the filter was fixed upside down with parafilm into a 2 ml collection tube, which was then placed into a 50 ml tube for stabilization during centrifugation. DNA was extracted from the Sterivex filter using the Qiagen DNeasy Blood & Tissue kit (Hilden, Germany). 20 μl proteinase K, 200 μl buffer AL and 220 μl PBS(-) were pipetted into each filter and incubated for 20 min at 56°C. eDNA was retrieved via centrifugation into a 2 ml collection tube. Subsequently, the standard protocol of the DNeasy kit was followed, eluting the extracted DNA in 200 μl AE buffer.

PCR amplification was performed with two consecutive PCRs. The first PCR included the gene specific primer sequences, tailed by a fragment of the standard Illumina adapter. The primers of the second PCR bound to the fragment of the introduced Illumina adapter, thereby completing the Illumina adapter required for sequencing and introducing sample specific indices required to de-multiplex in silico. We targeted a 316 bp fragment of mitochondrial cytochrome c oxidase subunit I (COI) using the mini-barcode primer combination BF1 (ACWGGWTGRACWGTNTAYCC) and BR2 (TCDGGRTGNCCRAARAAYCA [29]. To these primers we added six random nucleotides (to allow the removal of PCR duplicates during subsequent analyses) and the Illumina adapter fragment (for a complete list of full-length primers see S1 Table). Each sample was run with four replicates each to reduce PCR amplification biases. The PCR comprised 0.7 μl of each primer (10 mM), 5 μl 2x KAPPA HiHi HotStart ReadyMix (Roche), 1.6 μl ddH_2_O and 2 μl sample. The PCR ran at 95°C for 3 min, 35 cycles of 98°C for 10 s, 54°C for 15 s and 72°C for 15 s and a final elongation step at 72° for 5 min. In addition we targeted 12S rRNA using fish-specific MiFish primers [30]. However, during the computational analyses extensive cross-contaminations were detected in the 12S rRNA dataset, probably caused by a contamination of the 12S rRNA specific primers. Hence, we excluded the 12S rRNA data from the analyses.

After PCR, the four replicates per sample were mixed and cleaned with Ampliclean (Nimagen) magnetic beads following the instructions using 1.8× the volume of beads and eluting with 40 μl TE buffer. Concentrations of the PCR products were assessed on a Qubit 4 (Life Technologies, Singapore) and all were diluted to 0.1 ng/μl each prior to the second PCR. The second PCR comprised 0.3 μl of each primer (10 mM), 5 μl 2x KAPPA HiFi HotStart ReadyMix (Roche) and 4.4 μl of the diluted PCR product. The two-step PCR ran at 95°C for 3 min, 10 cycles of 98°C for 10 s and 72°C for 30 s and a final elongation step at 72°C for 5 min. Two additional negative controls (ddH_2_O instead of eDNA sample) were subjected to the same treatment with the two consecutive PCRs (to assess within lab cross contaminations) and were sequenced with the other samples. All samples and negative controls were pooled, the fragment length checked on a Tapestation (D1000 ScreenTape; Agilent) and the targeted gene fragment excised using BluePippin (1.5% dye free cassettes with marker L; Biozym), selecting the ‘tight’ option with a mean size of 527 bp to exclude primer dimers. The final concentration was determined prior to sequencing using Qubit 4. 60 μl of the pooled libraries with a concentration of 2 ng/μl were sent to Macrogen for 250 bp paired-end sequencing on a single MiSeq lane. Upon sequencing, Macrogen de-multiplexed the data based on the index combinations. PCR duplicates were removed using the “clone_filter” option in STACKS 2.5 [31] based on sequence identity and the random nucleotides introduced with the adapters. All sequences are deposited in the NCBI SRA repository (SAMN16484155- SAMN16484186; BioProject PRJNA669628; S1 Table). Low quality bases were cropped and complete primer and adapter sequences removed with Trim Galore (http://www.bioinformatics.babraham.ac.uk/projects/trim_galore/).

### Bioinformatic analyses

We employed and compared two different approaches for analyzing the eDNA data: 1) a series of short scripts to assemble OTUs and assign species via BLAST searches on NCBI (in the following referred to as the BLAST approach) and 2) using the mBRAVE pipeline [32].

To assemble OTUs for the BLAST approach, we followed in parts the methodology laid out in [33] (see S1 File for a detailed list of all employed steps and commands). All replicates were treated independently. Read pairs were merged with usearch 11.0.667 [34] and then de-replicated with vsearch version 2 [35], keeping only one copy of each unique sequence per replicate, while keeping the count. These were combined across all samples and then clustered into OTUs using a 3% threshold with usearch. Each unique OTU was locally blasted against NCBI’s nt database. Detailed taxonomic information was retrieved from NCBI via gb.accession2taxid and added to each OTU resulting in a master list with complete taxonomic information for each OTU. This information was then added to the OTUs present in each single replicate and all replicates concatenated into a single data sheet.

mBRAVE (the Multiplex Barcode Research And Visualization Environment) is a cloud-based data storage and bioinformatic platform with standardized pipelines and a sophisticated web interface for transforming raw HTS data into biological insights [32]. mBRAVE is developed by the team behind BOLD, and integrates common bioinformatic analytical methods and directly links to BOLD reference libraries [36], presenting users with the ability to analyze large volumes of HTS data, without requiring special technical training. The mBRAVE webserver is an important development and likely represents a landmark shift in standard DNA barcoding protocols. As per selection of available COI sequence libraries, we added 22 datasets including all SYS libraries (i.e., System Reference Library for mBRAVE ID Engine, Bacteria COI, Chordata, Fungi COI, Insecta, Non-Arthropoda Invertebrates, Non-Insect Arthropoda, Protista COI, Human Contamination Check, Standard Contaminants Based on Reagent Production), as well as additional 13 GBOL datasets representing 1,077,511, 693,911, 321,609, sequences, BINs and species, respectively.

The resulting OTUs and BINs from BLAST and mBRAVE were further cleaned to remove potential contaminations. mBRAVE already performs a range of analyses, however, potential contaminations were still included at this stage. First of all, we limited our analyses only to Metazoa and removed all non-metazoan OTUs. We further removed all potential contaminations. As potential contaminants we treated all OTUs with only two or less reads for a specific replicate (potentially due to leakage between replicates) or if this particular OTU was also present in one of the negative controls. In the latter case, we retained the OTU in the respective replicate only if its frequency was >10× the frequency observed in the negative controls. We further removed all OTUs with NCBI hits suggesting that they are non-COI (e.g., having 12S rRNA or small-subunit or similar in the sequence header). Six fish OTUs, which were present only in mBRAVE, were actually 12S rRNA sequences, which apparently have been misclassified as COI in BOLD. These are probably the result of cross-contamination (i.e., leakage) between libraries during sequencing and were removed from further analyses. In its final output, mBRAVE includes only OTUs which have a clear species identification (i.e., BIN from the BOLD database). In some instances more than one species name has been assigned to a BIN (e.g., due to identification errors or because species share highly similar sequences). In these instances, we selected the one most appropriate for the Elbe estuary, at least for fish species. NCBI-based BLAST results included numerous OTUs with low quality hits. Depending on the quality of the hit, we treated these either as “unknown Metazoa” or assigned them to a higher taxonomic category (e.g., Hexapoda, Mammalia, etc.) only, but removing their proposed species identification. Only OTUs with sequence similarities >97% and e-values >e^-50^ retained full species identifications; those with sequence similarities between 85–97% and e-values between e^-20^ and e^-50^ were assigned to their respective higher taxonomic categories and all others were treated as “unknown Metazoa” (in the following we will not distinguish between OTUs and BINs and treat all as OTUs).

In order to visualize the relationships of species composition at different stations we performed multi-variate statistical analyses in PAST v. 2.04 [37]. We performed principal component analyses (PCA) based on data grouped by stations (at stations B and C, the tidal levels were also considered). Data was coded as presence- absence for each OTU separately for the BLAST and mBRAVE datasets. We visualized the first two PC axes representing the majority of variation in the dataset. Further, we performed cluster analyses based on Euclidian distances as implemented in PAST in order to provide a hierarchical visualization of the data.

## Results

**Fig 2:**
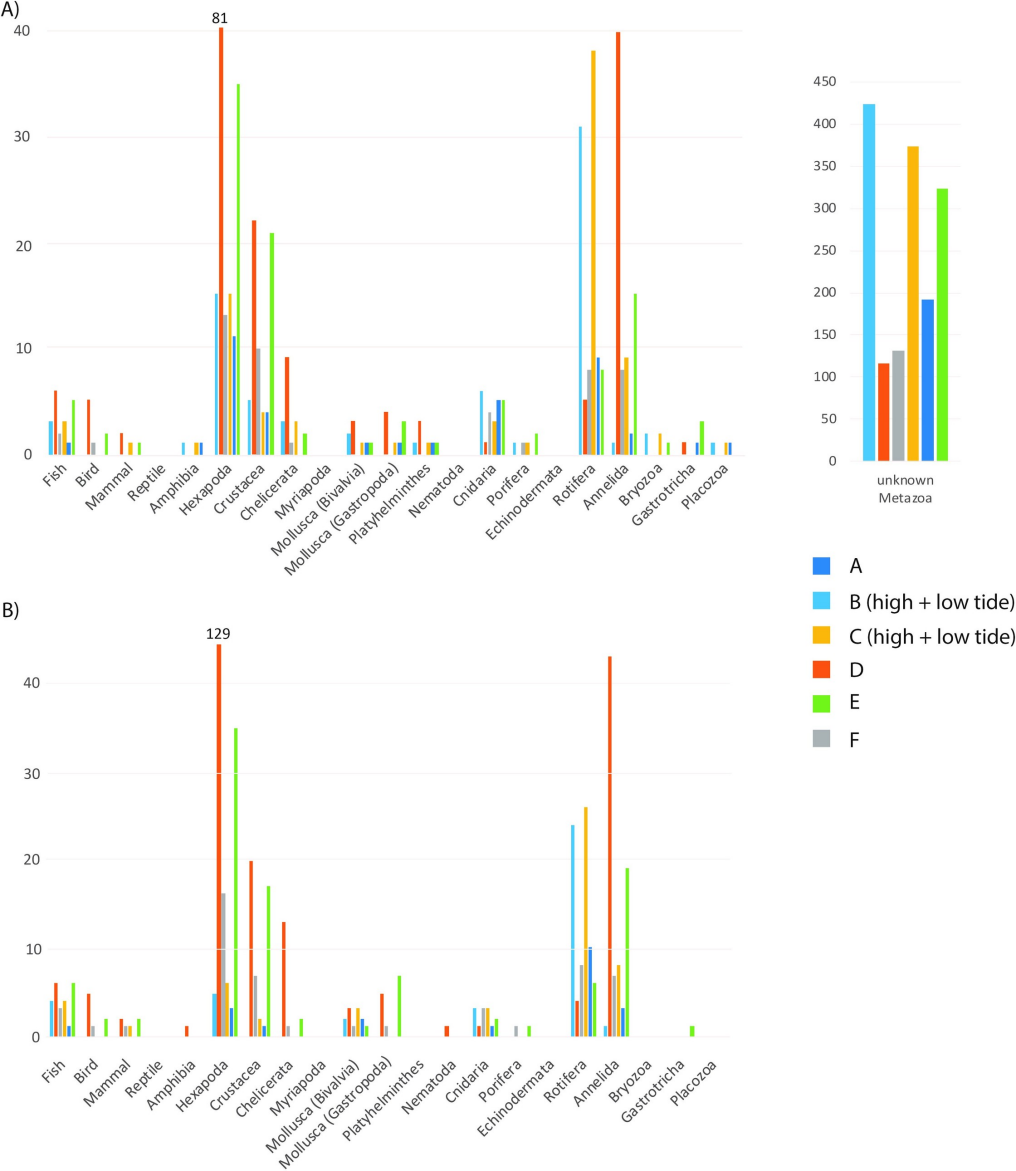
Total number of OTUs and BINs in A) BLAST and B) mBRAVE analyses recorded at each station. OTUs classified as “unknown Metazoa” are depicted separately due to the high numbers. Low and high tide data is merged for the respective stations. To improve readability, the bars representing Hexapoda at Station D (Lake Eichbaum) were shortened, the respective number of OTUs are shown above the bars. Stations A-C Elbe estuary, Station D Lake Eichbaum, Stations E and F Lake Hohendeich.

The BLAST approach yielded a total of 1294 OTUs, of which 214 (16.5%) had species level identifications, another 138 (10.7%) had identifications to higher taxonomic levels and 942 (72.8%) were not assigned to any taxon (classified as “unknown Metazoa”) following our strict filtering procedure (Fig 2; S1 and S2 Tables). The mBRAVE approach resulted in 355 OTUs, all with species identifications suggested by BOLD (Fig 2), though in several instances identifications referred to several closely related species (S1 and S3 Tables). In both analyses, most identified OTUs belonged to Hexapoda, followed by Annelida, Rotifera and Crustacea (Fig 2; S2 and S3 Tables). Noteworthy is the low number of mollusks, with less than ten bivalve and gastropod species, respectively.

The majority of all sequenced reads were of non-Metazoa and thus excluded from further analyses. In the BLAST-based results, non-Metazoa received twice as many (2.01×) reads compared to Metazoa (S1 Table). Though mBRAVE resulted in a higher fraction of metazoan reads, this is largely due to the overall low number of reads assigned to non-Metazoa (S1 Table). This likely is a result of the characteristics of the BOLD database in which taxa other than Metazoa are not identified by COI, but by other genetic markers. The total number of metazoan reads is relatively similar in BLAST (1,086,194 reads all Metazoa; 598,891 reads without “unknown Metazoa”) and mBRAVE (624,287 reads), though differs markedly in individual replicates (S1 Table). In the majority of replicates (with the Elbe station A being the sole exception), one or few OTUs made up an exceptionally high share of the respective reads (S1 Table). At the Elbe Stations B and C these were mainly Rotifera or “unknown Metazoa”, at Lake Eichbaum (Station D) Arthropoda and at Lake Hohendeich (Stations E and F) “unknown Metazoa” (S1–S3 Tables). For example, Rotifera comprised ~2/3 of all metazoan reads across all six replicates of Station C.

Filtering more water had no obvious impact on the number of recovered OTUs. Two of three replicates with more filtered water (1000 ml instead of 200 ml) had increased numbers of recovered OTUs, but here also the overall read numbers were higher potentially explaining the higher diversity (S2 and S3 Tables). The higher read number is probably a technical artefact from the pooling procedure as it was attempted to pool all samples at equal molarity, which would result in equal read numbers.

**Fig 3:**
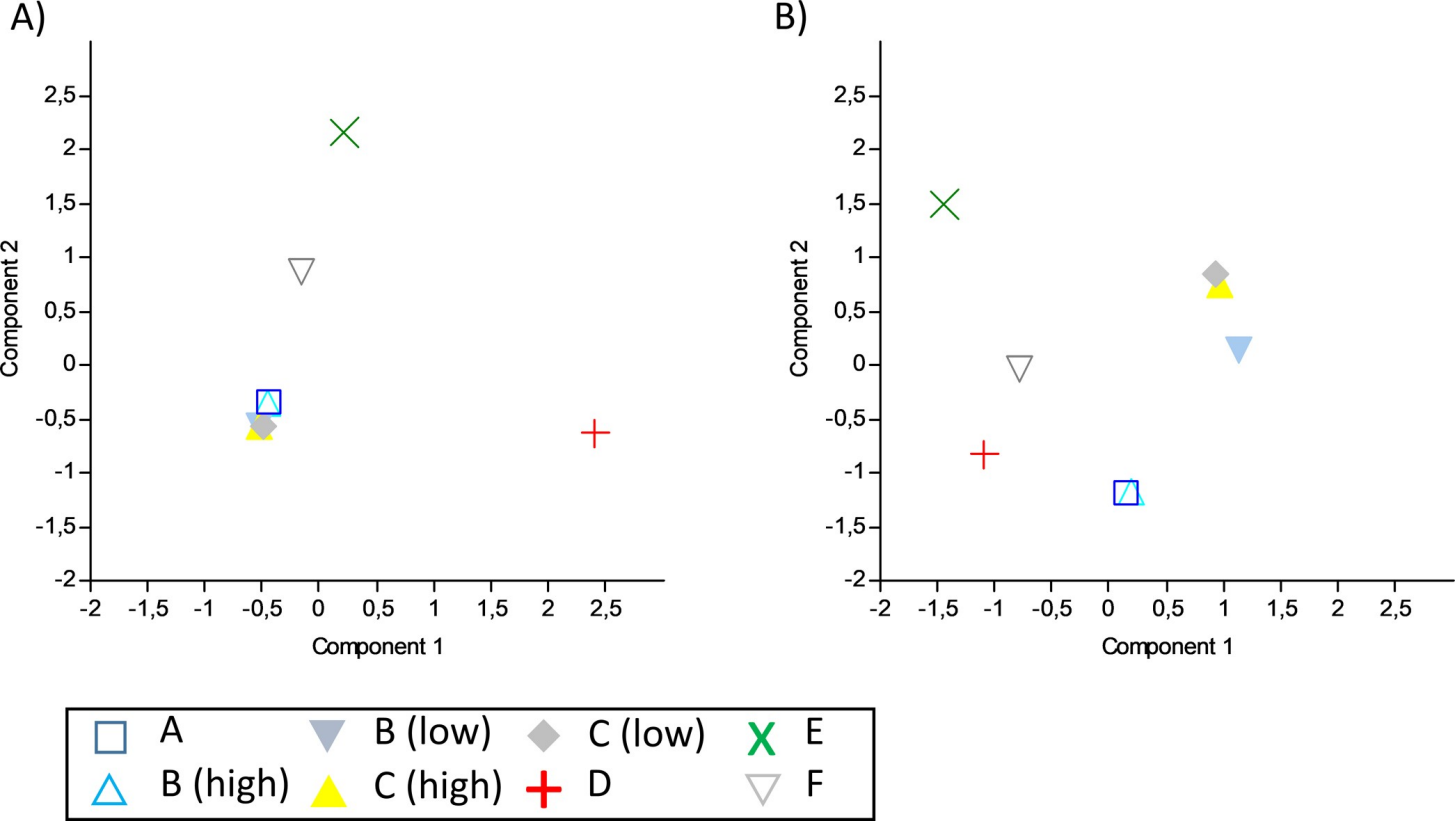
Principal component analyses (PCA) of all stations for A) mBRAVE and B) BLAST results. High and low tide sampling events are depicted separately for the respective stations. Stations A-C Elbe estuary, Station D Lake Eichbaum, Stations E and F Lake Hohendeich.

**Fig 4:**
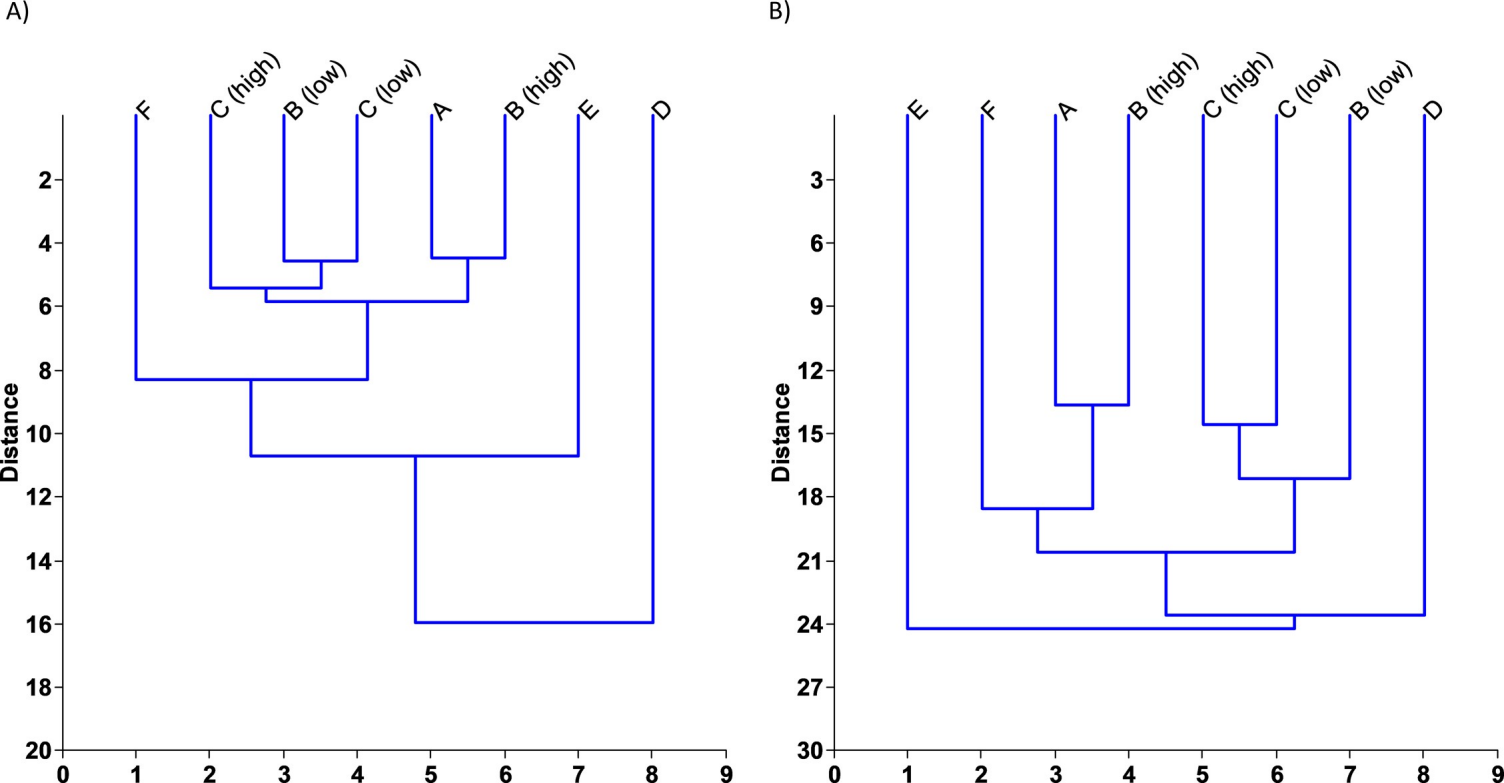
Cluster analysis of the OTUs retrieved in the A) mBRAVE and B) BLAST results. High and low tide sampling events are depicted separately for the respective stations. Stations A-C Elbe estuary, Station D Lake Eichbaum, Stations E and F Lake Hohendeich.

The replicates taken at each station either formed distinct groups in the cluster analyses and PCAs or grouped with replicates of stations with highly similar conditions (e.g., the two stations of Lake Hohendeich or of the Elbe estuary), suggesting that replicates are highly similar to each other. At the station-level, lake stations were clearly separated from each other (including both stations form Lake Hohendeich) and from the Elbe stations (Figs 3 and 4). The Elbe stations fell into two groups, one including the upstream station (Station C) at high and low tide and the downstream station (Station B) at low tide; the other group included Station B at high tide and Station A (also downstream) at early rising tide (1.5 hours after low tide; Figs 3 and 4; S1 Table). Thus, grouping of the estuary stations and sampling events was probably affected by tidal effects.

**Fig 5:**
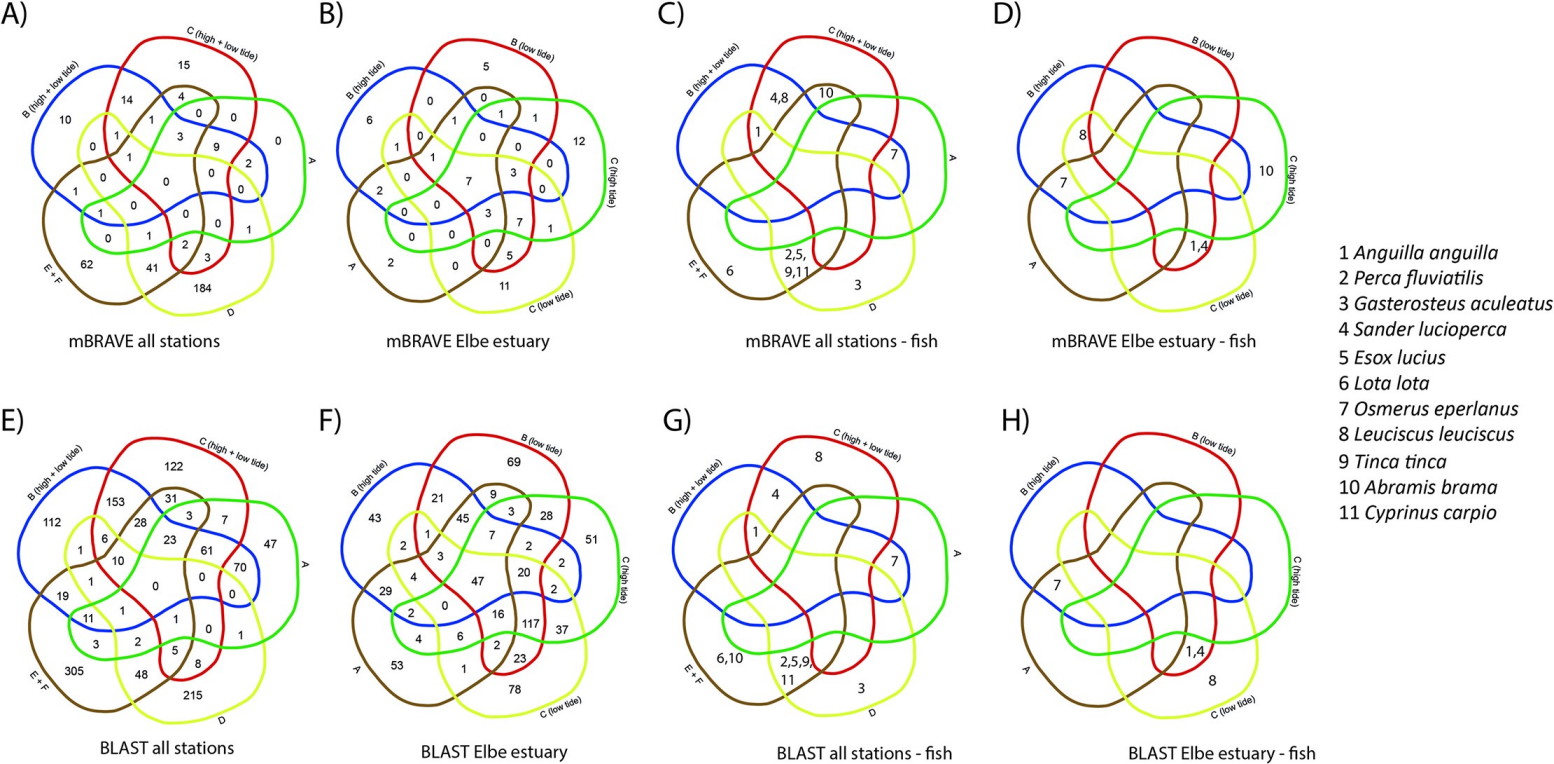
Venn diagrams of OTUs shared between stations. A–D) represent mBRAVE and E–H) BLAST results. A) and E) all Metazoa in all Elbe and lake stations (high and low tide sampling events combined), B) and F) all Metazoa in all Elbe stations with high and low tide events shown separately, C) and G) fish species in all Elbe and lake stations (high and low tide sampling events combined) and D) and H) fish species in all Elbe stations with high and low tide events shown separately. A), B), E), and F) show the number of shared OTUs, whereas in C), D), G) and H) shared fish species are indicated (here each number corresponds to a specific fish species as shown in the legend).

The majority of OTUs were recorded at a single station only (Fig 5): 271 of 355 OTUs for mBRAVE and 810 of 1294 OTUs for BLAST data (S2 and S3 Tables). Most of these were recorded at lake stations. Of the OTUs that were shared between stations, most were shared either among lakes (41 and 48 OTUs for mBRAVE and BLAST) or sets of the Elbe estuary stations (2, 9 and 14 OTUs for mBRAVE and 70, 61 and 153 for BLAST). Only 17 and 222 OTUs, respectively, were shared among any set of lake or Elbe River station for mBRAVE or BLAST analyses, none among all stations (Fig 5). Among Elbe River stations and sampling events, the majority of OTUs were recorded once only. Larger counts of shared OTUs were recorded when downstream stations at high (Station B) or early rising tide (Station A) were excluded (7 mBRAVE, 117 BLAST) and in BLAST, when only these two were considered in various combinations with the downstream station (Station B) at low tide (21, 25 and 25 OTUs, respectively) (Fig 5).

A detailed look at the OTUs identified as fish, suggests that only a few fish species were successfully sequenced (Fig 2; S2 and S3 Tables). Most fish species were recorded either at the estuary or lake stations, but not at both (Fig 5). Six fish species were recorded in each of the lakes, but only four among all Elbe River stations following BLAST (mBRAVE recovered one additional OTU in the Elbe River and one less for Lake Hohendeich).

## Discussion

In this study, we sampled and sequenced eDNA from several sites of the estuary of the Elbe and adjacent lakes to assess the power of eDNA to monitor fish and biodiversity in general in a well-mixed, turbid estuarine system. We found that only a low percentage of the fish species known from local fish assessments, performed with traditional catch devices, could be recovered in our sequence data. However, we also show that eDNA may be useful to monitor species of specific importance for the ecosystem, such as smelt and eel. We further recover a large number of invertebrates with our approach; many species for which so far only limited data have been available for the river system. Hence, the eDNA approach, with some modifications, may be highly valuable to get more detailed insights into the invertebrate fauna of large, aquatic ecosystems. Our results further showed a tidal effect on the recovery of species via eDNA at downriver stations, which might hint tide-dependent introduction of eDNA originating further upstream. Such effects need to be taken into consideration when applying eDNA to such systems. In the following, we discuss our findings in more detail.

### Tidal effects on the presence of eDNA and fauna shared between Elbe River and lakes

The freshwater section of the Elbe estuary reaches from the weir at Geesthacht downstream to about Elbe-km 655 including all stations studied herein. The water residence time in the tidal Elbe ranges from 2 to 12 weeks depending on discharge. Species living in the water column move with the water body approximately 15–20 km up- or downstream between high and low tides [38]. It should be pointed out that the tidal waters with their suspended matter in the studied section of the Elbe estuary are largely of riverine and not marine origin. Our data shows a clear and strong tidal influence on the recovered species. During low tide, the downstream station was highly similar to the upstream stations, suggesting that eDNA originating further upstream in the Elbe River dominated. This is not surprising as eDNA has been shown to travel > 10 km downstream from its source, though the transport distance appears to be species specific [20, 39, 40]. During early rising tide and high tide, the eDNA profile changed strongly at downriver station, whereas such a change was not observable at the upstream station. Due to our sampling design, these results are preliminary and to be considered with certain caution. Nevertheless, this might indicate tide-dependent introduction of eDNA originating further upstream into the estuary and a potential buffering effect of the Hamburg harbor, which might affect the retention of such upstream-originating eDNA. These results highlight the importance to sample multiple, widely-spaced stations and to sample repeatedly during high and low tide events to fully assess the biodiversity of well-mixed estuaries like the Elbe estuary in and around Hamburg using eDNA. This may also help to discern eDNA introduced from upstream or downstream areas during tides. If eDNA is to be used in biodiversity assessments of the Elbe estuary in the future, it is important to better understand such effects on biodiversity assessments to optimize the choice of localities and the timing of sampling.

### Species diversity revealed by eDNA

Diversity assessments by eDNA are biased on the one hand by the relative abundance and availability of eDNA, which may differ between taxa [20], and potential biases during PCR amplification. For the latter, the specificity of the utilized primers is a crucial factor [41]. The COI primer pair used herein was tested on invertebrate mock communities, where it had shown high recovery rates [29]. This is also mirrored in our data with large numbers of invertebrates of various taxonomic groups being successfully sequenced and recovered. The relatively low number of mollusks is probably not a reflection of their true diversity, but may be due to the selected primers [26].

The known fish fauna of the tidal freshwater section of the Elbe estuary of the territory of the city of Hamburg contains 49 species [42], the fish faunas of Lake Eichbaum and Lake Hohendeich comprise 17 and 14 species, respectively [43]. Those species numbers have been recorded with classic catch methods over many years. Based on the results obtained with the BLAST pipeline (and very similar for the mBRAVE pipeline), with our eDNA approach we were able to detect only four species in the Elbe estuary. Three of these–*Anguilla anguilla*, *Sander lucioperca* and *Osmerus eperlanus*–belong to the ten most common fish species in these stretches of the Elbe estuary [42]. This corresponds to a relatively low recovery rate of the known fish fauna via eDNA of 8.2% for the Elbe estuary. Lake Eichbaum and Lake Hohendeich yielded six species each with eDNA, resulting in higher recovery rates for the lakes: 35.3% for Lake Eichbaum and 42.9% for Lake Hohendeich. The fish fauna of both lakes was relatively well represented, in particular as our sampling points were few (one or two) and exclusively in shallow water, very close to the water edge. By comparison, the known fish fauna of the Elbe estuary was barely recovered with eDNA. This seems to be not an exceptional case, but a common pattern in eDNA approaches when compared to more classical monitoring techniques [44, 45]. We cannot be sure of the reasons for this. One possible explanation could be the relatively small quantities of filtered water (200 ml per replicate), which may have favored species/individuals that were in direct proximity to our sampling sites. Furthermore, the distribution of reads among taxa suggests that the broad range of sequenced taxa in combination with the strong dominance of single OTUs (representing the majority of reads in many replicates) reduced the resolution for species with lower eDNA yields. One main advantage of using universal primers–the ability to target virtually all taxa–resulted in a dataset that comprised twice as many non-metazoan than metazoan reads, which is disadvantageous if the focus is set on Metazoa. The use of less degenerate primers may overcome this problem [45]. OTUs with disproportional high read depth could be either due to preferential binding of primers (appears less likely here), a higher amount of available eDNA, or because whole individuals or parts thereof were captured during eDNA sampling. For the Elbe estuary samples, with the high abundance of the small-bodied Rotifera, the latter appears particularly likely. When the Elbe estuary samples were collected, the filters were quickly clogged by suspended aggregates, which are present in high numbers in the Elbe waters. Maybe Rotifera (as well as bacteria) were attached to these estuarine aggregates, and were therefore preferentially included in our DNA libraries.

While we showed that the recovery rates strongly differ between flowing and standing water for the fish communities, we did not have similar reference data for other taxa. Nevertheless, we recovered a relatively high diversity for many invertebrate groups. One interesting pattern found in the insect data is that especially many Chironomidae were recovered. Data for Diptera in general are sparse and aquatic eDNA studies may help, especially in understudied species groups to generate important distribution data.

### Comparison between analytical pipelines

At first glance, analyzing the sequencing data with our BLAST pipeline recovered a much higher number of potential species (OTUs) than the mBRAVE approach. However, the difference is largely due to the more than 900 metazoan OTUs without clear identifications present in the BLAST results. These are missing for mBRAVE, as here only OTUs that can be assigned to species (or to BINs more precisely) are included. The number of OTUs recovered for each higher taxon were similar, but not identical. For example, 37 additional hexapod OTUs were identified by mBRAVE and 17 more rotifers by BLAST. Again, some of these smaller discrepancies can be due to OTUs that we classified as unknown Metazoa in the BLAST approach, but may have been assigned to a specific OTU by mBRAVE or vice versa. Employing more than one analytical strategy may help to identify potentially problematic taxonomic assignments or other sources of potential biases inherent in the respective strategy. For example, the presence of taxonomically unassigned OTUs may help to identify overall patterns of diversity, as well as putative needs to improve reference databases, but may be unwanted in assessments of particular bioindicators. As the COI barcode inventory grows continuously, the number of unidentified OTUs will decrease in the future and the gap between the BLAST and mBRAVE pipelines will (probably) close further. Until then it may depend on the specific goals, which analytical strategy is more appropriate. If it is important to include only OTUs that can be assigned to a specific species or higher metazoan taxon either BOLD or BLAST with stringent filtering are appropriate. If unidentified metazoan species should be included as well to have a more complete assessment of local diversities a BLAST strategy is required. Similarly, a BLAST strategy is required if there is interest in non-metazoan diversity, as BOLD does not routinely store COI for non-Metazoa. However, one should be aware that COI does not yield the same resolution in several non-metazoan groups as for Metazoa.

An obvious drawback of mBRAVE was the often ambiguous species identification, which for example suggested various closely related fish species for each OTU (or BIN). This complicates detailed studies of local diversities, as multiple potentially co-occurring species would be summed under one OTU, and the detection of invasive species would be severely limited in cases where closely related species already inhabit the respective habitat.

## Supporting information

S1 File Detailed report of all the computational steps and analyses performed in the BLAST-based analysis. (DOCX)

S1 Table List of all sampled stations and replicates including information on ecological parameters during sampling, barcode sequences, and reads counts for various groups of taxa as well as total numbers of OTUs of the BLAST and mBRAVE analyses. For samples from the Elbe estuary, the tide during sampling as well as the closest high (HT) or low (LT) tide event is provided.
(XLSX)

S2 Table BLAST results. Statistics for each OTU from each replicate provided. For each OTU the number of reads per replicate is shown, as well as the GenBank accession number and further associated information of the closest hit in NCBI. Full taxonomic information is provided only for OTUs with sequence similarities >97% and e-values >e^-50^ to the respective closest hit, for sequence similarities between 85–97% and e-values between e^-20^ and e^-50^ only the respective higher taxonomic categories are provided and all others were assigned to “unknown Metazoa”.
(XLSX)

S3 Table mBRAVE results. Statistics for each OTU (= BIN) from each replicate provided as reported by mBRAVE.
(XLSX)
