# Supplementary material for: eDNA as a tool for non-invasive monitoring of the fauna of a turbid, well-mixed system, the Elbe estuary in Germany
Source: PLoS One. 2021 Apr 16;16(4):e0250452. doi: 10.1371/journal.pone.0250452 (PMC8051793; doi:10.1371/journal.pone.0250452)
Supplement: S1 File — (DOCX) [file pone.0250452.s001.docx]

## **Trimming with Trimgalore**

trim_galore --paired --quality 30 --length 140 ../raw_data/1_1_12S_1.fastq.gz ../raw_data/1_1_12S_2.fastq.gz

trim_galore --paired --quality 30 --length 140 ../raw_data/1_1_COI_1.fastq.gz ../raw_data/1_1_COI_2.fastq.gz

trim_galore --paired --quality 30 --length 140 ../raw_data/1_2_12S_1.fastq.gz ../raw_data/1_2_12S_2.fastq.gz

trim_galore --paired --quality 30 --length 140 ../raw_data/1_2_COI_1.fastq.gz ../raw_data/1_2_COI_2.fastq.gz

trim_galore --paired --quality 30 --length 140 ../raw_data/1_3_12S_1.fastq.gz ../raw_data/1_3_12S_2.fastq.gz

trim_galore --paired --quality 30 --length 140 ../raw_data/1_3_COI_1.fastq.gz ../raw_data/1_3_COI_2.fastq.gz

trim_galore --paired --quality 30 --length 140 ../raw_data/1_4_12S_1.fastq.gz ../raw_data/1_4_12S_2.fastq.gz

trim_galore --paired --quality 30 --length 140 ../raw_data/1_4_COI_1.fastq.gz ../raw_data/1_4_COI_2.fastq.gz

trim_galore --paired --quality 30 --length 140 ../raw_data/1_5_12S_1.fastq.gz ../raw_data/1_5_12S_2.fastq.gz

trim_galore --paired --quality 30 --length 140 ../raw_data/1_5_COI_1.fastq.gz ../raw_data/1_5_COI_2.fastq.gz

trim_galore --paired --quality 30 --length 140 ../raw_data/1_6_12S_1.fastq.gz ../raw_data/1_6_12S_2.fastq.gz

trim_galore --paired --quality 30 --length 140 ../raw_data/1_6_COI_1.fastq.gz ../raw_data/1_6_COI_2.fastq.gz

trim_galore --paired --quality 30 --length 140 ../raw_data/2_1a_12S_1.fastq.gz ../raw_data/2_1a_12S_2.fastq.gz

trim_galore --paired --quality 30 --length 140 ../raw_data/2_1a_COI_1.fastq.gz ../raw_data/2_1a_COI_2.fastq.gz

trim_galore --paired --quality 30 --length 140 ../raw_data/2_1b_12S_1.fastq.gz ../raw_data/2_1b_12S_2.fastq.gz

trim_galore --paired --quality 30 --length 140 ../raw_data/2_1b_COI_1.fastq.gz ../raw_data/2_1b_COI_2.fastq.gz

trim_galore --paired --quality 30 --length 140 ../raw_data/2_2_12S_1.fastq.gz ../raw_data/2_2_12S_2.fastq.gz

trim_galore --paired --quality 30 --length 140 ../raw_data/2_2_COI_1.fastq.gz ../raw_data/2_2_COI_2.fastq.gz

trim_galore --paired --quality 30 --length 140 ../raw_data/2_3_12S_1.fastq.gz ../raw_data/2_3_12S_2.fastq.gz

trim_galore --paired --quality 30 --length 140 ../raw_data/2_3_COI_1.fastq.gz ../raw_data/2_3_COI_2.fastq.gz

trim_galore --paired --quality 30 --length 140 ../raw_data/3_1_12S_1.fastq.gz ../raw_data/3_1_12S_2.fastq.gz

trim_galore --paired --quality 30 --length 140 ../raw_data/3_1_COI_1.fastq.gz ../raw_data/3_1_COI_2.fastq.gz

trim_galore --paired --quality 30 --length 140 ../raw_data/3_2a_12S_1.fastq.gz ../raw_data/3_2a_12S_2.fastq.gz

trim_galore --paired --quality 30 --length 140 ../raw_data/3_2a_COI_1.fastq.gz ../raw_data/3_2a_COI_2.fastq.gz

trim_galore --paired --quality 30 --length 140 ../raw_data/3_2b_12S_1.fastq.gz ../raw_data/3_2b_12S_2.fastq.gz

trim_galore --paired --quality 30 --length 140 ../raw_data/3_2b_COI_1.fastq.gz ../raw_data/3_2b_COI_2.fastq.gz

trim_galore --paired --quality 30 --length 140 ../raw_data/3_3_12S_1.fastq.gz ../raw_data/3_3_12S_2.fastq.gz

trim_galore --paired --quality 30 --length 140 ../raw_data/3_3_COI_1.fastq.gz ../raw_data/3_3_COI_2.fastq.gz

trim_galore --paired --quality 30 --length 140 ../raw_data/4_1_12S_1.fastq.gz ../raw_data/4_1_12S_2.fastq.gz

trim_galore --paired --quality 30 --length 140 ../raw_data/4_1_COI_1.fastq.gz ../raw_data/4_1_COI_2.fastq.gz

trim_galore --paired --quality 30 --length 140 ../raw_data/4_2_12S_1.fastq.gz ../raw_data/4_2_12S_2.fastq.gz

trim_galore --paired --quality 30 --length 140 ../raw_data/4_2_COI_1.fastq.gz ../raw_data/4_2_COI_2.fastq.gz

trim_galore --paired --quality 30 --length 140 ../raw_data/4_3_12S_1.fastq.gz ../raw_data/4_3_12S_2.fastq.gz

trim_galore --paired --quality 30 --length 140 ../raw_data/4_3_COI_1.fastq.gz ../raw_data/4_3_COI_2.fastq.gz

trim_galore --paired --quality 30 --length 140 ../raw_data/4_4_12S_1.fastq.gz ../raw_data/4_4_12S_2.fastq.gz

trim_galore --paired --quality 30 --length 140 ../raw_data/4_4_COI_1.fastq.gz ../raw_data/4_4_COI_2.fastq.gz

trim_galore --paired --quality 30 --length 140 ../raw_data/4_5_12S_1.fastq.gz ../raw_data/4_5_12S_2.fastq.gz

trim_galore --paired --quality 30 --length 140 ../raw_data/4_5_COI_1.fastq.gz ../raw_data/4_5_COI_2.fastq.gz

trim_galore --paired --quality 30 --length 140 ../raw_data/4_6_12S_1.fastq.gz ../raw_data/4_6_12S_2.fastq.gz

trim_galore --paired --quality 30 --length 140 ../raw_data/4_6_COI_1.fastq.gz ../raw_data/4_6_COI_2.fastq.gz

trim_galore --paired --quality 30 --length 140 ../raw_data/5_1_12S_1.fastq.gz ../raw_data/5_1_12S_2.fastq.gz

trim_galore --paired --quality 30 --length 140 ../raw_data/5_1_COI_1.fastq.gz ../raw_data/5_1_COI_2.fastq.gz

trim_galore --paired --quality 30 --length 140 ../raw_data/5_2_12S_1.fastq.gz ../raw_data/5_2_12S_2.fastq.gz

trim_galore --paired --quality 30 --length 140 ../raw_data/5_2_COI_1.fastq.gz ../raw_data/5_2_COI_2.fastq.gz

trim_galore --paired --quality 30 --length 140 ../raw_data/5_3_12S_1.fastq.gz ../raw_data/5_3_12S_2.fastq.gz

trim_galore --paired --quality 30 --length 140 ../raw_data/5_3_COI_1.fastq.gz ../raw_data/5_3_COI_2.fastq.gz

trim_galore --paired --quality 30 --length 140 ../raw_data/6_1a_12S_1.fastq.gz ../raw_data/6_1a_12S_2.fastq.gz

trim_galore --paired --quality 30 --length 140 ../raw_data/6_1a_COI_1.fastq.gz ../raw_data/6_1a_COI_2.fastq.gz

trim_galore --paired --quality 30 --length 140 ../raw_data/6_1b_12S_1.fastq.gz ../raw_data/6_1b_12S_2.fastq.gz

trim_galore --paired --quality 30 --length 140 ../raw_data/6_1b_COI_extracted_1.fastq.gz ../raw_data/6_1b_COI_extracted_2.fastq.gz

trim_galore --paired --quality 30 --length 140 ../raw_data/6_2_12S_1.fastq.gz ../raw_data/6_2_12S_2.fastq.gz

trim_galore --paired --quality 30 --length 140 ../raw_data/6_2_COI_extracted_1.fastq.gz ../raw_data/6_2_COI_extracted_2.fastq.gz

trim_galore --paired --quality 30 --length 140 ../raw_data/6_3_12S_1.fastq.gz ../raw_data/6_3_12S_2.fastq.gz

trim_galore --paired --quality 30 --length 140 ../raw_data/6_3_COI_1.fastq.gz ../raw_data/6_3_COI_2.fastq.gz

trim_galore --paired --quality 30 --length 140 ../raw_data/H2O-89A__12S_extracted_1.fastq.gz ../raw_data/H2O-89A__12S_extracted_2.fastq.gz

trim_galore --paired --quality 30 --length 140 ../raw_data/H2O-89B_12S_extracted_1.fastq.gz ../raw_data/H2O-89B_12S_extracted_2.fastq.gz

trim_galore --paired --quality 30 --length 140 ../raw_data/H2O-90A__COI_1.fastq.gz ../raw_data/H2O-90A__COI_2.fastq.gz

trim_galore --paired --quality 30 --length 140 ../raw_data/H2O90-B__COI_1.fastq.gz ../raw_data/H2O90-B__COI_2.fastq.gz

trim_galore --paired --quality 30 --length 140 ../raw_data/nega_ctrl_1_COI_1.fastq.gz ../raw_data/nega_ctrl_1_COI_2.fastq.gz

trim_galore --paired --quality 30 --length 140 ../raw_data/neg_ctrl_1_12S_1.fastq.gz ../raw_data/neg_ctrl_1_12S_2.fastq.gz

trim_galore --paired --quality 30 --length 140 ../raw_data/neg_ctrl_2_12S_1.fastq.gz ../raw_data/neg_ctrl_2_12S_2.fastq.gz

trim_galore --paired --quality 30 --length 140 ../raw_data/neg_ctrl_3_12S_1.fastq.gz ../raw_data/neg_ctrl_3_12S_2.fastq.gz

trim_galore --paired --quality 30 --length 140 ../raw_data/neg_ctrl_3_COI_1.fastq.gz ../raw_data/neg_ctrl_3_COI_2.fastq.gz

trim_galore --paired --quality 30 --length 140 ../raw_data/neg_ctrll_2_COI_1.fastq.gz ../raw_data/neg_ctrll_2_COI_2.fastq.gz

## **remove PCR duplicates using Stacks**

also used to remove Ns and primer (COI: 6 Ns plus 20 bp primer; 12 S forward: 6 Ns plus 22 bp Primer; 12S reverse 6Ns plus 28 bp primer)

clone_filter -1 ./1_1_COI_1_val_1.fq.gz -2 ./1_1_COI_2_val_2.fq.gz -i gzfastq -o ./filtered/ --inline_inline --oligo_len_1 26 --oligo_len_2 26

clone_filter -1 ./1_2_COI_1_val_1.fq.gz -2 ./1_2_COI_2_val_2.fq.gz -i gzfastq -o ./filtered/ --inline_inline --oligo_len_1 26 --oligo_len_2 26

clone_filter -1 ./1_3_COI_1_val_1.fq.gz -2 ./1_3_COI_2_val_2.fq.gz -i gzfastq -o ./filtered/ --inline_inline --oligo_len_1 26 --oligo_len_2 26

clone_filter -1 ./1_4_COI_1_val_1.fq.gz -2 ./1_4_COI_2_val_2.fq.gz -i gzfastq -o ./filtered/ --inline_inline --oligo_len_1 26 --oligo_len_2 26

clone_filter -1 ./1_5_COI_1_val_1.fq.gz -2 ./1_5_COI_2_val_2.fq.gz -i gzfastq -o ./filtered/ --inline_inline --oligo_len_1 26 --oligo_len_2 26

clone_filter -1 ./1_6_COI_1_val_1.fq.gz -2 ./1_6_COI_2_val_2.fq.gz -i gzfastq -o ./filtered/ --inline_inline --oligo_len_1 26 --oligo_len_2 26

clone_filter -1 ./2_1a_COI_1_val_1.fq.gz -2 ./2_1a_COI_2_val_2.fq.gz -i gzfastq -o ./filtered/ --inline_inline --oligo_len_1 26 --oligo_len_2 26

clone_filter -1 ./2_1b_COI_1_val_1.fq.gz -2 ./2_1b_COI_2_val_2.fq.gz -i gzfastq -o ./filtered/ --inline_inline --oligo_len_1 26 --oligo_len_2 26

clone_filter -1 ./2_2_COI_1_val_1.fq.gz -2 ./2_2_COI_2_val_2.fq.gz -i gzfastq -o ./filtered/ --inline_inline --oligo_len_1 26 --oligo_len_2 26

clone_filter -1 ./2_3_COI_1_val_1.fq.gz -2 ./2_3_COI_2_val_2.fq.gz -i gzfastq -o ./filtered/ --inline_inline --oligo_len_1 26 --oligo_len_2 26

clone_filter -1 ./3_1_COI_1_val_1.fq.gz -2 ./3_1_COI_2_val_2.fq.gz -i gzfastq -o ./filtered/ --inline_inline --oligo_len_1 26 --oligo_len_2 26

clone_filter -1 ./3_2a_COI_1_val_1.fq.gz -2 ./3_2a_COI_2_val_2.fq.gz -i gzfastq -o ./filtered/ --inline_inline --oligo_len_1 26 --oligo_len_2 26

clone_filter -1 ./3_2b_COI_1_val_1.fq.gz -2 ./3_2b_COI_2_val_2.fq.gz -i gzfastq -o ./filtered/ --inline_inline --oligo_len_1 26 --oligo_len_2 26

clone_filter -1 ./3_3_COI_1_val_1.fq.gz -2 ./3_3_COI_2_val_2.fq.gz -i gzfastq -o ./filtered/ --inline_inline --oligo_len_1 26 --oligo_len_2 26

clone_filter -1 ./4_1_COI_1_val_1.fq.gz -2 ./4_1_COI_2_val_2.fq.gz -i gzfastq -o ./filtered/ --inline_inline --oligo_len_1 26 --oligo_len_2 26

clone_filter -1 ./4_2_COI_1_val_1.fq.gz -2 ./4_2_COI_2_val_2.fq.gz -i gzfastq -o ./filtered/ --inline_inline --oligo_len_1 26 --oligo_len_2 26

clone_filter -1 ./4_3_COI_1_val_1.fq.gz -2 ./4_3_COI_2_val_2.fq.gz -i gzfastq -o ./filtered/ --inline_inline --oligo_len_1 26 --oligo_len_2 26

clone_filter -1 ./4_4_COI_1_val_1.fq.gz -2 ./4_4_COI_2_val_2.fq.gz -i gzfastq -o ./filtered/ --inline_inline --oligo_len_1 26 --oligo_len_2 26

clone_filter -1 ./4_5_COI_1_val_1.fq.gz -2 ./4_5_COI_2_val_2.fq.gz -i gzfastq -o ./filtered/ --inline_inline --oligo_len_1 26 --oligo_len_2 26

clone_filter -1 ./4_6_COI_1_val_1.fq.gz -2 ./4_6_COI_2_val_2.fq.gz -i gzfastq -o ./filtered/ --inline_inline --oligo_len_1 26 --oligo_len_2 26

clone_filter -1 ./5_1_COI_1_val_1.fq.gz -2 ./5_1_COI_2_val_2.fq.gz -i gzfastq -o ./filtered/ --inline_inline --oligo_len_1 26 --oligo_len_2 26

clone_filter -1 ./5_2_COI_1_val_1.fq.gz -2 ./5_2_COI_2_val_2.fq.gz -i gzfastq -o ./filtered/ --inline_inline --oligo_len_1 26 --oligo_len_2 26

clone_filter -1 ./5_3_COI_1_val_1.fq.gz -2 ./5_3_COI_2_val_2.fq.gz -i gzfastq -o ./filtered/ --inline_inline --oligo_len_1 26 --oligo_len_2 26

clone_filter -1 ./6_1a_COI_1_val_1.fq.gz -2 ./6_1a_COI_2_val_2.fq.gz -i gzfastq -o ./filtered/ --inline_inline --oligo_len_1 26 --oligo_len_2 26

clone_filter -1 ./6_1b_COI_extracted_1_val_1.fq.gz -2 ./6_1b_COI_extracted_2_val_2.fq.gz -i gzfastq -o ./filtered/ --inline_inline --oligo_len_1 26 --oligo_len_2 26

clone_filter -1 ./6_2_COI_extracted_1_val_1.fq.gz -2 ./6_2_COI_extracted_2_val_2.fq.gz -i gzfastq -o ./filtered/ --inline_inline --oligo_len_1 26 --oligo_len_2 26

clone_filter -1 ./6_3_COI_1_val_1.fq.gz -2 ./6_3_COI_2_val_2.fq.gz -i gzfastq -o ./filtered/ --inline_inline --oligo_len_1 26 --oligo_len_2 26

clone_filter -1 ./H2O-90A__COI_1_val_1.fq.gz -2 ./H2O-90A__COI_2_val_2.fq.gz -i gzfastq -o ./filtered/ --inline_inline --oligo_len_1 26 --oligo_len_2 26

clone_filter -1 ./H2O90-B__COI_1_val_1.fq.gz -2 ./H2O90-B__COI_2_val_2.fq.gz -i gzfastq -o ./filtered/ --inline_inline --oligo_len_1 26 --oligo_len_2 26

clone_filter -1 ./nega_ctrl_1_COI_1_val_1.fq.gz -2 ./nega_ctrl_1_COI_2_val_2.fq.gz -i gzfastq -o ./filtered/ --inline_inline --oligo_len_1 26 --oligo_len_2 26

clone_filter -1 ./neg_ctrl_3_COI_1_val_1.fq.gz -2 ./neg_ctrl_3_COI_2_val_2.fq.gz -i gzfastq -o ./filtered/ --inline_inline --oligo_len_1 26 --oligo_len_2 26

clone_filter -1 ./neg_ctrll_2_COI_1_val_1.fq.gz -2 ./neg_ctrll_2_COI_2_val_2.fq.gz -i gzfastq -o ./filtered/ --inline_inline --oligo_len_1 26 --oligo_len_2 26

clone_filter -1 ./1_1_12S_1_val_1.fq.gz -2 ./1_1_12S_2_val_2.fq.gz -i gzfastq -o ./filtered/ --inline_inline --oligo_len_1 28 --oligo_len_2 34

clone_filter -1 ./1_2_12S_1_val_1.fq.gz -2 ./1_2_12S_2_val_2.fq.gz -i gzfastq -o ./filtered/ --inline_inline --oligo_len_1 28 --oligo_len_2 34

clone_filter -1 ./1_3_12S_1_val_1.fq.gz -2 ./1_3_12S_2_val_2.fq.gz -i gzfastq -o ./filtered/ --inline_inline --oligo_len_1 28 --oligo_len_2 34

clone_filter -1 ./1_4_12S_1_val_1.fq.gz -2 ./1_4_12S_2_val_2.fq.gz -i gzfastq -o ./filtered/ --inline_inline --oligo_len_1 28 --oligo_len_2 34

clone_filter -1 ./1_5_12S_1_val_1.fq.gz -2 ./1_5_12S_2_val_2.fq.gz -i gzfastq -o ./filtered/ --inline_inline --oligo_len_1 28 --oligo_len_2 34

clone_filter -1 ./1_6_12S_1_val_1.fq.gz -2 ./1_6_12S_2_val_2.fq.gz -i gzfastq -o ./filtered/ --inline_inline --oligo_len_1 28 --oligo_len_2 34

clone_filter -1 ./2_1a_12S_1_val_1.fq.gz -2 ./2_1a_12S_2_val_2.fq.gz -i gzfastq -o ./filtered/ --inline_inline --oligo_len_1 28 --oligo_len_2 34

clone_filter -1 ./2_1b_12S_1_val_1.fq.gz -2 ./2_1b_12S_2_val_2.fq.gz -i gzfastq -o ./filtered/ --inline_inline --oligo_len_1 28 --oligo_len_2 34

clone_filter -1 ./2_2_12S_1_val_1.fq.gz -2 ./2_2_12S_2_val_2.fq.gz -i gzfastq -o ./filtered/ --inline_inline --oligo_len_1 28 --oligo_len_2 34

clone_filter -1 ./2_3_12S_1_val_1.fq.gz -2 ./2_3_12S_2_val_2.fq.gz -i gzfastq -o ./filtered/ --inline_inline --oligo_len_1 28 --oligo_len_2 34

clone_filter -1 ./3_1_12S_1_val_1.fq.gz -2 ./3_1_12S_2_val_2.fq.gz -i gzfastq -o ./filtered/ --inline_inline --oligo_len_1 28 --oligo_len_2 34

clone_filter -1 ./3_2a_12S_1_val_1.fq.gz -2 ./3_2a_12S_2_val_2.fq.gz -i gzfastq -o ./filtered/ --inline_inline --oligo_len_1 28 --oligo_len_2 34

clone_filter -1 ./3_2b_12S_1_val_1.fq.gz -2 ./3_2b_12S_2_val_2.fq.gz -i gzfastq -o ./filtered/ --inline_inline --oligo_len_1 28 --oligo_len_2 34

clone_filter -1 ./3_3_12S_1_val_1.fq.gz -2 ./3_3_12S_2_val_2.fq.gz -i gzfastq -o ./filtered/ --inline_inline --oligo_len_1 28 --oligo_len_2 34

clone_filter -1 ./4_1_12S_1_val_1.fq.gz -2 ./4_1_12S_2_val_2.fq.gz -i gzfastq -o ./filtered/ --inline_inline --oligo_len_1 28 --oligo_len_2 34

clone_filter -1 ./4_2_12S_1_val_1.fq.gz -2 ./4_2_12S_2_val_2.fq.gz -i gzfastq -o ./filtered/ --inline_inline --oligo_len_1 28 --oligo_len_2 34

clone_filter -1 ./4_3_12S_1_val_1.fq.gz -2 ./4_3_12S_2_val_2.fq.gz -i gzfastq -o ./filtered/ --inline_inline --oligo_len_1 28 --oligo_len_2 34

clone_filter -1 ./4_4_12S_1_val_1.fq.gz -2 ./4_4_12S_2_val_2.fq.gz -i gzfastq -o ./filtered/ --inline_inline --oligo_len_1 28 --oligo_len_2 34

clone_filter -1 ./4_5_12S_1_val_1.fq.gz -2 ./4_5_12S_2_val_2.fq.gz -i gzfastq -o ./filtered/ --inline_inline --oligo_len_1 28 --oligo_len_2 34

clone_filter -1 ./4_6_12S_1_val_1.fq.gz -2 ./4_6_12S_2_val_2.fq.gz -i gzfastq -o ./filtered/ --inline_inline --oligo_len_1 28 --oligo_len_2 34

clone_filter -1 ./5_1_12S_1_val_1.fq.gz -2 ./5_1_12S_2_val_2.fq.gz -i gzfastq -o ./filtered/ --inline_inline --oligo_len_1 28 --oligo_len_2 34

clone_filter -1 ./5_2_12S_1_val_1.fq.gz -2 ./5_2_12S_2_val_2.fq.gz -i gzfastq -o ./filtered/ --inline_inline --oligo_len_1 28 --oligo_len_2 34

clone_filter -1 ./5_3_12S_1_val_1.fq.gz -2 ./5_3_12S_2_val_2.fq.gz -i gzfastq -o ./filtered/ --inline_inline --oligo_len_1 28 --oligo_len_2 34

clone_filter -1 ./6_1a_12S_1_val_1.fq.gz -2 ./6_1a_12S_2_val_2.fq.gz -i gzfastq -o ./filtered/ --inline_inline --oligo_len_1 28 --oligo_len_2 34

clone_filter -1 ./6_1b_12S_1_val_1.fq.gz -2 ./6_1b_12S_2_val_2.fq.gz -i gzfastq -o ./filtered/ --inline_inline --oligo_len_1 28 --oligo_len_2 34

clone_filter -1 ./6_2_12S_1_val_1.fq.gz -2 ./6_2_12S_2_val_2.fq.gz -i gzfastq -o ./filtered/ --inline_inline --oligo_len_1 28 --oligo_len_2 34

clone_filter -1 ./6_3_12S_1_val_1.fq.gz -2 ./6_3_12S_2_val_2.fq.gz -i gzfastq -o ./filtered/ --inline_inline --oligo_len_1 28 --oligo_len_2 34

clone_filter -1 ./H2O-89A__12S_extracted_1_val_1.fq.gz -2 ./H2O-89A__12S_extracted_2_val_2.fq.gz -i gzfastq -o ./filtered/ --inline_inline --oligo_len_1 28 --oligo_len_2 34

clone_filter -1 ./H2O-89B_12S_extracted_1_val_1.fq.gz -2 ./H2O-89B_12S_extracted_2_val_2.fq.gz -i gzfastq -o ./filtered/ --inline_inline --oligo_len_1 28 --oligo_len_2 34

clone_filter -1 ./neg_ctrl_1_12S_1_val_1.fq.gz -2 ./neg_ctrl_1_12S_2_val_2.fq.gz -i gzfastq -o ./filtered/ --inline_inline --oligo_len_1 28 --oligo_len_2 34

clone_filter -1 ./neg_ctrl_2_12S_1_val_1.fq.gz -2 ./neg_ctrl_2_12S_2_val_2.fq.gz -i gzfastq -o ./filtered/ --inline_inline --oligo_len_1 28 --oligo_len_2 34

clone_filter -1 ./neg_ctrl_3_12S_1_val_1.fq.gz -2 ./neg_ctrl_3_12S_2_val_2.fq.gz -i gzfastq -o ./filtered/ --inline_inline --oligo_len_1 28 --oligo_len_2 34

## **merge read pairs with usearch**

/home/f6nv175/usearch11.0.667 -fastq_mergepairs 1_1_12S_1_val_1.1.fq -reverse 1_1_12S_2_val_2.2.fq -fastaout 1_1_12S_assembled.fq

/home/f6nv175/usearch11.0.667 -fastq_mergepairs 1_1_COI_1_val_1.1.fq -reverse 1_1_COI_2_val_2.2.fq -fastaout 1_1_COI_assembled.fq

/home/f6nv175/usearch11.0.667 -fastq_mergepairs 1_2_12S_1_val_1.1.fq -reverse 1_2_12S_2_val_2.2.fq -fastaout 1_2_12S_assembled.fq

/home/f6nv175/usearch11.0.667 -fastq_mergepairs 1_2_COI_1_val_1.1.fq -reverse 1_2_COI_2_val_2.2.fq -fastaout 1_2_COI_assembled.fq

/home/f6nv175/usearch11.0.667 -fastq_mergepairs 1_3_12S_1_val_1.1.fq -reverse 1_3_12S_2_val_2.2.fq -fastaout 1_3_12S_assembled.fq

/home/f6nv175/usearch11.0.667 -fastq_mergepairs 1_3_COI_1_val_1.1.fq -reverse 1_3_COI_2_val_2.2.fq -fastaout 1_3_COI_assembled.fq

/home/f6nv175/usearch11.0.667 -fastq_mergepairs 1_4_12S_1_val_1.1.fq -reverse 1_4_12S_2_val_2.2.fq -fastaout 1_4_12S_assembled.fq

/home/f6nv175/usearch11.0.667 -fastq_mergepairs 1_4_COI_1_val_1.1.fq -reverse 1_4_COI_2_val_2.2.fq -fastaout 1_4_COI_assembled.fq

/home/f6nv175/usearch11.0.667 -fastq_mergepairs 1_5_12S_1_val_1.1.fq -reverse 1_5_12S_2_val_2.2.fq -fastaout 1_5_12S_assembled.fq

/home/f6nv175/usearch11.0.667 -fastq_mergepairs 1_5_COI_1_val_1.1.fq -reverse 1_5_COI_2_val_2.2.fq -fastaout 1_5_COI_assembled.fq

/home/f6nv175/usearch11.0.667 -fastq_mergepairs 1_6_12S_1_val_1.1.fq -reverse 1_6_12S_2_val_2.2.fq -fastaout 1_6_12S_assembled.fq

/home/f6nv175/usearch11.0.667 -fastq_mergepairs 1_6_COI_1_val_1.1.fq -reverse 1_6_COI_2_val_2.2.fq -fastaout 1_6_COI_assembled.fq

/home/f6nv175/usearch11.0.667 -fastq_mergepairs 2_1a_12S_1_val_1.1.fq -reverse 2_1a_12S_2_val_2.2.fq -fastaout 2_1a_12S_assembled.fq

/home/f6nv175/usearch11.0.667 -fastq_mergepairs 2_1a_COI_1_val_1.1.fq -reverse 2_1a_COI_2_val_2.2.fq -fastaout 2_1a_COI_assembled.fq

/home/f6nv175/usearch11.0.667 -fastq_mergepairs 2_1b_12S_1_val_1.1.fq -reverse 2_1b_12S_2_val_2.2.fq -fastaout 2_1b_12S_assembled.fq

/home/f6nv175/usearch11.0.667 -fastq_mergepairs 2_1b_COI_1_val_1.1.fq -reverse 2_1b_COI_2_val_2.2.fq -fastaout 2_1b_COI_assembled.fq

/home/f6nv175/usearch11.0.667 -fastq_mergepairs 2_2_12S_1_val_1.1.fq -reverse 2_2_12S_2_val_2.2.fq -fastaout 2_2_12S_assembled.fq

/home/f6nv175/usearch11.0.667 -fastq_mergepairs 2_2_COI_1_val_1.1.fq -reverse 2_2_COI_2_val_2.2.fq -fastaout 2_2_COI_assembled.fq

/home/f6nv175/usearch11.0.667 -fastq_mergepairs 2_3_12S_1_val_1.1.fq -reverse 2_3_12S_2_val_2.2.fq -fastaout 2_3_12S_assembled.fq

/home/f6nv175/usearch11.0.667 -fastq_mergepairs 2_3_COI_1_val_1.1.fq -reverse 2_3_COI_2_val_2.2.fq -fastaout 2_3_COI_assembled.fq

/home/f6nv175/usearch11.0.667 -fastq_mergepairs 3_1_12S_1_val_1.1.fq -reverse 3_1_12S_2_val_2.2.fq -fastaout 3_1_12S_assembled.fq

/home/f6nv175/usearch11.0.667 -fastq_mergepairs 3_1_COI_1_val_1.1.fq -reverse 3_1_COI_2_val_2.2.fq -fastaout 3_1_COI_assembled.fq

/home/f6nv175/usearch11.0.667 -fastq_mergepairs 3_2a_12S_1_val_1.1.fq -reverse 3_2a_12S_2_val_2.2.fq -fastaout 3_2a_12S_assembled.fq

/home/f6nv175/usearch11.0.667 -fastq_mergepairs 3_2a_COI_1_val_1.1.fq -reverse 3_2a_COI_2_val_2.2.fq -fastaout 3_2a_COI_assembled.fq

/home/f6nv175/usearch11.0.667 -fastq_mergepairs 3_2b_12S_1_val_1.1.fq -reverse 3_2b_12S_2_val_2.2.fq -fastaout 3_2b_12S_assembled.fq

/home/f6nv175/usearch11.0.667 -fastq_mergepairs 3_2b_COI_1_val_1.1.fq -reverse 3_2b_COI_2_val_2.2.fq -fastaout 3_2b_COI_assembled.fq

/home/f6nv175/usearch11.0.667 -fastq_mergepairs 3_3_12S_1_val_1.1.fq -reverse 3_3_12S_2_val_2.2.fq -fastaout 3_3_12S_assembled.fq

/home/f6nv175/usearch11.0.667 -fastq_mergepairs 3_3_COI_1_val_1.1.fq -reverse 3_3_COI_2_val_2.2.fq -fastaout 3_3_COI_assembled.fq

/home/f6nv175/usearch11.0.667 -fastq_mergepairs 4_1_12S_1_val_1.1.fq -reverse 4_1_12S_2_val_2.2.fq -fastaout 4_1_12S_assembled.fq

/home/f6nv175/usearch11.0.667 -fastq_mergepairs 4_1_COI_1_val_1.1.fq -reverse 4_1_COI_2_val_2.2.fq -fastaout 4_1_COI_assembled.fq

/home/f6nv175/usearch11.0.667 -fastq_mergepairs 4_2_12S_1_val_1.1.fq -reverse 4_2_12S_2_val_2.2.fq -fastaout 4_2_12S_assembled.fq

/home/f6nv175/usearch11.0.667 -fastq_mergepairs 4_2_COI_1_val_1.1.fq -reverse 4_2_COI_2_val_2.2.fq -fastaout 4_2_COI_assembled.fq

/home/f6nv175/usearch11.0.667 -fastq_mergepairs 4_3_12S_1_val_1.1.fq -reverse 4_3_12S_2_val_2.2.fq -fastaout 4_3_12S_assembled.fq

/home/f6nv175/usearch11.0.667 -fastq_mergepairs 4_3_COI_1_val_1.1.fq -reverse 4_3_COI_2_val_2.2.fq -fastaout 4_3_COI_assembled.fq

/home/f6nv175/usearch11.0.667 -fastq_mergepairs 4_4_12S_1_val_1.1.fq -reverse 4_4_12S_2_val_2.2.fq -fastaout 4_4_12S_assembled.fq

/home/f6nv175/usearch11.0.667 -fastq_mergepairs 4_4_COI_1_val_1.1.fq -reverse 4_4_COI_2_val_2.2.fq -fastaout 4_4_COI_assembled.fq

/home/f6nv175/usearch11.0.667 -fastq_mergepairs 4_5_12S_1_val_1.1.fq -reverse 4_5_12S_2_val_2.2.fq -fastaout 4_5_12S_assembled.fq

/home/f6nv175/usearch11.0.667 -fastq_mergepairs 4_5_COI_1_val_1.1.fq -reverse 4_5_COI_2_val_2.2.fq -fastaout 4_5_COI_assembled.fq

/home/f6nv175/usearch11.0.667 -fastq_mergepairs 4_6_12S_1_val_1.1.fq -reverse 4_6_12S_2_val_2.2.fq -fastaout 4_6_12S_assembled.fq

/home/f6nv175/usearch11.0.667 -fastq_mergepairs 4_6_COI_1_val_1.1.fq -reverse 4_6_COI_2_val_2.2.fq -fastaout 4_6_COI_assembled.fq

/home/f6nv175/usearch11.0.667 -fastq_mergepairs 5_1_12S_1_val_1.1.fq -reverse 5_1_12S_2_val_2.2.fq -fastaout 5_1_12S_assembled.fq

/home/f6nv175/usearch11.0.667 -fastq_mergepairs 5_1_COI_1_val_1.1.fq -reverse 5_1_COI_2_val_2.2.fq -fastaout 5_1_COI_assembled.fq

/home/f6nv175/usearch11.0.667 -fastq_mergepairs 5_2_12S_1_val_1.1.fq -reverse 5_2_12S_2_val_2.2.fq -fastaout 5_2_12S_assembled.fq

/home/f6nv175/usearch11.0.667 -fastq_mergepairs 5_2_COI_1_val_1.1.fq -reverse 5_2_COI_2_val_2.2.fq -fastaout 5_2_COI_assembled.fq

/home/f6nv175/usearch11.0.667 -fastq_mergepairs 5_3_12S_1_val_1.1.fq -reverse 5_3_12S_2_val_2.2.fq -fastaout 5_3_12S_assembled.fq

/home/f6nv175/usearch11.0.667 -fastq_mergepairs 5_3_COI_1_val_1.1.fq -reverse 5_3_COI_2_val_2.2.fq -fastaout 5_3_COI_assembled.fq

/home/f6nv175/usearch11.0.667 -fastq_mergepairs 6_1a_12S_1_val_1.1.fq -reverse 6_1a_12S_2_val_2.2.fq -fastaout 6_1a_12S_assembled.fq

/home/f6nv175/usearch11.0.667 -fastq_mergepairs 6_1a_COI_1_val_1.1.fq -reverse 6_1a_COI_2_val_2.2.fq -fastaout 6_1a_COI_assembled.fq

/home/f6nv175/usearch11.0.667 -fastq_mergepairs 6_1b_12S_1_val_1.1.fq -reverse 6_1b_12S_2_val_2.2.fq -fastaout 6_1b_12S_assembled.fq

/home/f6nv175/usearch11.0.667 -fastq_mergepairs 6_1b_COI_extracted_1_val_1.1.fq -reverse 6_1b_COI_extracted_2_val_2.2.fq -fastaout 6_1b_COI_extracted_assembled.fq

/home/f6nv175/usearch11.0.667 -fastq_mergepairs 6_2_12S_1_val_1.1.fq -reverse 6_2_12S_2_val_2.2.fq -fastaout 6_2_12S_assembled.fq

/home/f6nv175/usearch11.0.667 -fastq_mergepairs 6_2_COI_extracted_1_val_1.1.fq -reverse 6_2_COI_extracted_2_val_2.2.fq -fastaout 6_2_COI_extracted_assembled.fq

/home/f6nv175/usearch11.0.667 -fastq_mergepairs 6_3_12S_1_val_1.1.fq -reverse 6_3_12S_2_val_2.2.fq -fastaout 6_3_12S_assembled.fq

/home/f6nv175/usearch11.0.667 -fastq_mergepairs 6_3_COI_1_val_1.1.fq -reverse 6_3_COI_2_val_2.2.fq -fastaout 6_3_COI_assembled.fq

/home/f6nv175/usearch11.0.667 -fastq_mergepairs H2O-89A__12S_extracted_1_val_1.1.fq -reverse H2O-89A__12S_extracted_2_val_2.2.fq -fastaout H2O-89A__12S_extracted_assembled.fq

/home/f6nv175/usearch11.0.667 -fastq_mergepairs H2O-89B_12S_extracted_1_val_1.1.fq -reverse H2O-89B_12S_extracted_2_val_2.2.fq -fastaout H2O-89B_12S_extracted_assembled.fq

/home/f6nv175/usearch11.0.667 -fastq_mergepairs H2O-90A__COI_1_val_1.1.fq -reverse H2O-90A__COI_2_val_2.2.fq -fastaout H2O-90A__COI_assembled.fq

/home/f6nv175/usearch11.0.667 -fastq_mergepairs H2O90-B__COI_1_val_1.1.fq -reverse H2O90-B__COI_2_val_2.2.fq -fastaout H2O90-B__COI_assembled.fq

/home/f6nv175/usearch11.0.667 -fastq_mergepairs nega_ctrl_1_COI_1_val_1.1.fq -reverse nega_ctrl_1_COI_2_val_2.2.fq -fastaout nega_ctrl_1_COI_assembled.fq

/home/f6nv175/usearch11.0.667 -fastq_mergepairs neg_ctrl_1_12S_1_val_1.1.fq -reverse neg_ctrl_1_12S_2_val_2.2.fq -fastaout neg_ctrl_1_12S_assembled.fq

/home/f6nv175/usearch11.0.667 -fastq_mergepairs neg_ctrl_2_12S_1_val_1.1.fq -reverse neg_ctrl_2_12S_2_val_2.2.fq -fastaout neg_ctrl_2_12S_assembled.fq

/home/f6nv175/usearch11.0.667 -fastq_mergepairs neg_ctrl_3_12S_1_val_1.1.fq -reverse neg_ctrl_3_12S_2_val_2.2.fq -fastaout neg_ctrl_3_12S_assembled.fq

/home/f6nv175/usearch11.0.667 -fastq_mergepairs neg_ctrl_3_COI_1_val_1.1.fq -reverse neg_ctrl_3_COI_2_val_2.2.fq -fastaout neg_ctrl_3_COI_assembled.fq

/home/f6nv175/usearch11.0.667 -fastq_mergepairs neg_ctrll_2_COI_1_val_1.1.fq -reverse neg_ctrll_2_COI_2_val_2.2.fq -fastaout neg_ctrll_2_COI_assembled.fq

## **4. dereplicate with vsearch (keeping only one copy of each sequence, and keeping the count!)**

vsearch -derep_fulllength 1_1_12S_assembled.fq -output 1_1_12S_assembled_count.fq -sizeout

vsearch -derep_fulllength 1_1_COI_assembled.fq -output 1_1_COI_assembled_count.fq -sizeout

vsearch -derep_fulllength 1_2_12S_assembled.fq -output 1_2_12S_assembled_count.fq -sizeout

vsearch -derep_fulllength 1_2_COI_assembled.fq -output 1_2_COI_assembled_count.fq -sizeout

vsearch -derep_fulllength 1_3_12S_assembled.fq -output 1_3_12S_assembled_count.fq -sizeout

vsearch -derep_fulllength 1_3_COI_assembled.fq -output 1_3_COI_assembled_count.fq -sizeout

vsearch -derep_fulllength 1_4_12S_assembled.fq -output 1_4_12S_assembled_count.fq -sizeout

vsearch -derep_fulllength 1_4_COI_assembled.fq -output 1_4_COI_assembled_count.fq -sizeout

vsearch -derep_fulllength 1_5_12S_assembled.fq -output 1_5_12S_assembled_count.fq -sizeout

vsearch -derep_fulllength 1_5_COI_assembled.fq -output 1_5_COI_assembled_count.fq -sizeout

vsearch -derep_fulllength 1_6_12S_assembled.fq -output 1_6_12S_assembled_count.fq -sizeout

vsearch -derep_fulllength 1_6_COI_assembled.fq -output 1_6_COI_assembled_count.fq -sizeout

vsearch -derep_fulllength 2_1a_12S_assembled.fq -output 2_1a_12S_assembled_count.fq -sizeout

vsearch -derep_fulllength 2_1a_COI_assembled.fq -output 2_1a_COI_assembled_count.fq -sizeout

vsearch -derep_fulllength 2_1b_12S_assembled.fq -output 2_1b_12S_assembled_count.fq -sizeout

vsearch -derep_fulllength 2_1b_COI_assembled.fq -output 2_1b_COI_assembled_count.fq -sizeout

vsearch -derep_fulllength 2_2_12S_assembled.fq -output 2_2_12S_assembled_count.fq -sizeout

vsearch -derep_fulllength 2_2_COI_assembled.fq -output 2_2_COI_assembled_count.fq -sizeout

vsearch -derep_fulllength 2_3_12S_assembled.fq -output 2_3_12S_assembled_count.fq -sizeout

vsearch -derep_fulllength 2_3_COI_assembled.fq -output 2_3_COI_assembled_count.fq -sizeout

vsearch -derep_fulllength 3_1_12S_assembled.fq -output 3_1_12S_assembled_count.fq -sizeout

vsearch -derep_fulllength 3_1_COI_assembled.fq -output 3_1_COI_assembled_count.fq -sizeout

vsearch -derep_fulllength 3_2a_12S_assembled.fq -output 3_2a_12S_assembled_count.fq -sizeout

vsearch -derep_fulllength 3_2a_COI_assembled.fq -output 3_2a_COI_assembled_count.fq -sizeout

vsearch -derep_fulllength 3_2b_12S_assembled.fq -output 3_2b_12S_assembled_count.fq -sizeout

vsearch -derep_fulllength 3_2b_COI_assembled.fq -output 3_2b_COI_assembled_count.fq -sizeout

vsearch -derep_fulllength 3_3_12S_assembled.fq -output 3_3_12S_assembled_count.fq -sizeout

vsearch -derep_fulllength 3_3_COI_assembled.fq -output 3_3_COI_assembled_count.fq -sizeout

vsearch -derep_fulllength 4_1_12S_assembled.fq -output 4_1_12S_assembled_count.fq -sizeout

vsearch -derep_fulllength 4_1_COI_assembled.fq -output 4_1_COI_assembled_count.fq -sizeout

vsearch -derep_fulllength 4_2_12S_assembled.fq -output 4_2_12S_assembled_count.fq -sizeout

vsearch -derep_fulllength 4_2_COI_assembled.fq -output 4_2_COI_assembled_count.fq -sizeout

vsearch -derep_fulllength 4_3_12S_assembled.fq -output 4_3_12S_assembled_count.fq -sizeout

vsearch -derep_fulllength 4_3_COI_assembled.fq -output 4_3_COI_assembled_count.fq -sizeout

vsearch -derep_fulllength 4_4_12S_assembled.fq -output 4_4_12S_assembled_count.fq -sizeout

vsearch -derep_fulllength 4_4_COI_assembled.fq -output 4_4_COI_assembled_count.fq -sizeout

vsearch -derep_fulllength 4_5_12S_assembled.fq -output 4_5_12S_assembled_count.fq -sizeout

vsearch -derep_fulllength 4_5_COI_assembled.fq -output 4_5_COI_assembled_count.fq -sizeout

vsearch -derep_fulllength 4_6_12S_assembled.fq -output 4_6_12S_assembled_count.fq -sizeout

vsearch -derep_fulllength 4_6_COI_assembled.fq -output 4_6_COI_assembled_count.fq -sizeout

vsearch -derep_fulllength 5_1_12S_assembled.fq -output 5_1_12S_assembled_count.fq -sizeout

vsearch -derep_fulllength 5_1_COI_assembled.fq -output 5_1_COI_assembled_count.fq -sizeout

vsearch -derep_fulllength 5_2_12S_assembled.fq -output 5_2_12S_assembled_count.fq -sizeout

vsearch -derep_fulllength 5_2_COI_assembled.fq -output 5_2_COI_assembled_count.fq -sizeout

vsearch -derep_fulllength 5_3_12S_assembled.fq -output 5_3_12S_assembled_count.fq -sizeout

vsearch -derep_fulllength 5_3_COI_assembled.fq -output 5_3_COI_assembled_count.fq -sizeout

vsearch -derep_fulllength 6_1a_12S_assembled.fq -output 6_1a_12S_assembled_count.fq -sizeout

vsearch -derep_fulllength 6_1a_COI_assembled.fq -output 6_1a_COI_assembled_count.fq -sizeout

vsearch -derep_fulllength 6_1b_12S_assembled.fq -output 6_1b_12S_assembled_count.fq -sizeout

vsearch -derep_fulllength 6_1b_COI_extracted_assembled.fq -output 6_1b_COI_extracted_assembled_count.fq -sizeout

vsearch -derep_fulllength 6_2_12S_assembled.fq -output 6_2_12S_assembled_count.fq -sizeout

vsearch -derep_fulllength 6_2_COI_extracted_assembled.fq -output 6_2_COI_extracted_assembled_count.fq -sizeout

vsearch -derep_fulllength 6_3_12S_assembled.fq -output 6_3_12S_assembled_count.fq -sizeout

vsearch -derep_fulllength 6_3_COI_assembled.fq -output 6_3_COI_assembled_count.fq -sizeout

vsearch -derep_fulllength H2O-89A__12S_extracted_assembled.fq -output H2O-89A__12S_extracted_assembled_count.fq -sizeout

vsearch -derep_fulllength H2O-89B_12S_extracted_assembled.fq -output H2O-89B_12S_extracted_assembled_count.fq -sizeout

vsearch -derep_fulllength H2O-90A__COI_assembled.fq -output H2O-90A__COI_assembled_count.fq -sizeout

vsearch -derep_fulllength H2O90-B__COI_assembled.fq -output H2O90-B__COI_assembled_count.fq -sizeout

vsearch -derep_fulllength nega_ctrl_1_COI_assembled.fq -output nega_ctrl_1_COI_assembled_count.fq -sizeout

vsearch -derep_fulllength neg_ctrl_1_12S_assembled.fq -output neg_ctrl_1_12S_assembled_count.fq -sizeout

vsearch -derep_fulllength neg_ctrl_2_12S_assembled.fq -output neg_ctrl_2_12S_assembled_count.fq -sizeout

vsearch -derep_fulllength neg_ctrl_3_12S_assembled.fq -output neg_ctrl_3_12S_assembled_count.fq -sizeout

vsearch -derep_fulllength neg_ctrl_3_COI_assembled.fq -output neg_ctrl_3_COI_assembled_count.fq -sizeout

vsearch -derep_fulllength neg_ctrll_2_COI_assembled.fq -output neg_ctrll_2_COI_assembled_count.fq -sizeout

## **5. create “master” for OTU creation (adding to pre-existing count file)**

concatenate all derepliacated/counted files of each gene

- concatenating the two newly assembled files with all previously assembled files

cat *COI_*.fq > COI_all_OTUs_2020-06-09.txt

dereplicate the pooled files with sizeout + sizein to keep read abundances! Use minuniquesize 5 to remove singletons and potential contaminations!

vsearch -derep_fulllength COI_all_sequences_2020-06-09.txt -output COI_all_sequences_count_derep_min5_2020-06-09.fq -sizein -sizeout -minuniquesize 5

## **6) cluster OTUs mit usearch**

Cluster with 3% similarity on both strands!

/home/f6nv175/usearch11.0.667 -cluster_otus COI_all_sequences_count_derep_min5_2020-06-09.fq -otus COI_all_OTUs_2020-06-09.txt -uparseout COI_all_OTU_out_table_2020-06-09.txt -relabel OTU_ -strand both

compare dereplicated reads of each replica against the OTUs without chimeras (repeated for 12S with 99% clustering threshold

/home/f6nv175/usearch11.0.667 -usearch_global 1_1_COI_assembled_count.fq -db COI_all_OTUs_2020-06-09.txt -strand both -id 0.97 -maxhits 1 -blast6out 1_1_COI_OTUs.txt

/home/f6nv175/usearch11.0.667 -usearch_global 1_2_COI_assembled_count.fq -db COI_all_OTUs_2020-06-09.txt -strand both -id 0.97 -maxhits 1 -blast6out 1_2_COI_OTUs.txt

/home/f6nv175/usearch11.0.667 -usearch_global 1_3_COI_assembled_count.fq -db COI_all_OTUs_2020-06-09.txt -strand both -id 0.97 -maxhits 1 -blast6out 1_3_COI_OTUs.txt

/home/f6nv175/usearch11.0.667 -usearch_global 1_4_COI_assembled_count.fq -db COI_all_OTUs_2020-06-09.txt -strand both -id 0.97 -maxhits 1 -blast6out 1_4_COI_OTUs.txt

/home/f6nv175/usearch11.0.667 -usearch_global 1_5_COI_assembled_count.fq -db COI_all_OTUs_2020-06-09.txt -strand both -id 0.97 -maxhits 1 -blast6out 1_5_COI_OTUs.txt

/home/f6nv175/usearch11.0.667 -usearch_global 1_6_COI_assembled_count.fq -db COI_all_OTUs_2020-06-09.txt -strand both -id 0.97 -maxhits 1 -blast6out 1_6_COI_OTUs.txt

/home/f6nv175/usearch11.0.667 -usearch_global 2_1a_COI_assembled_count.fq -db COI_all_OTUs_2020-06-09.txt -strand both -id 0.97 -maxhits 1 -blast6out 2_1a_COI_OTUs.txt

/home/f6nv175/usearch11.0.667 -usearch_global 2_1b_COI_assembled_count.fq -db COI_all_OTUs_2020-06-09.txt -strand both -id 0.97 -maxhits 1 -blast6out 2_1b_COI_OTUs.txt

/home/f6nv175/usearch11.0.667 -usearch_global 2_2_COI_assembled_count.fq -db COI_all_OTUs_2020-06-09.txt -strand both -id 0.97 -maxhits 1 -blast6out 2_2_COI_OTUs.txt

/home/f6nv175/usearch11.0.667 -usearch_global 2_3_COI_assembled_count.fq -db COI_all_OTUs_2020-06-09.txt -strand both -id 0.97 -maxhits 1 -blast6out 2_3_COI_OTUs.txt

/home/f6nv175/usearch11.0.667 -usearch_global 3_1_COI_assembled_count.fq -db COI_all_OTUs_2020-06-09.txt -strand both -id 0.97 -maxhits 1 -blast6out 3_1_COI_OTUs.txt

/home/f6nv175/usearch11.0.667 -usearch_global 3_2a_COI_assembled_count.fq -db COI_all_OTUs_2020-06-09.txt -strand both -id 0.97 -maxhits 1 -blast6out 3_2a_COI_OTUs.txt

/home/f6nv175/usearch11.0.667 -usearch_global 3_2b_COI_assembled_count.fq -db COI_all_OTUs_2020-06-09.txt -strand both -id 0.97 -maxhits 1 -blast6out 3_2b_COI_OTUs.txt

/home/f6nv175/usearch11.0.667 -usearch_global 3_3_COI_assembled_count.fq -db COI_all_OTUs_2020-06-09.txt -strand both -id 0.97 -maxhits 1 -blast6out 3_3_COI_OTUs.txt

/home/f6nv175/usearch11.0.667 -usearch_global 4_1_COI_assembled_count.fq -db COI_all_OTUs_2020-06-09.txt -strand both -id 0.97 -maxhits 1 -blast6out 4_1_COI_OTUs.txt

/home/f6nv175/usearch11.0.667 -usearch_global 4_2_COI_assembled_count.fq -db COI_all_OTUs_2020-06-09.txt -strand both -id 0.97 -maxhits 1 -blast6out 4_2_COI_OTUs.txt

/home/f6nv175/usearch11.0.667 -usearch_global 4_3_COI_assembled_count.fq -db COI_all_OTUs_2020-06-09.txt -strand both -id 0.97 -maxhits 1 -blast6out 4_3_COI_OTUs.txt

/home/f6nv175/usearch11.0.667 -usearch_global 4_4_COI_assembled_count.fq -db COI_all_OTUs_2020-06-09.txt -strand both -id 0.97 -maxhits 1 -blast6out 4_4_COI_OTUs.txt

/home/f6nv175/usearch11.0.667 -usearch_global 4_5_COI_assembled_count.fq -db COI_all_OTUs_2020-06-09.txt -strand both -id 0.97 -maxhits 1 -blast6out 4_5_COI_OTUs.txt

/home/f6nv175/usearch11.0.667 -usearch_global 4_6_COI_assembled_count.fq -db COI_all_OTUs_2020-06-09.txt -strand both -id 0.97 -maxhits 1 -blast6out 4_6_COI_OTUs.txt

/home/f6nv175/usearch11.0.667 -usearch_global 5_1_COI_assembled_count.fq -db COI_all_OTUs_2020-06-09.txt -strand both -id 0.97 -maxhits 1 -blast6out 5_1_COI_OTUs.txt

/home/f6nv175/usearch11.0.667 -usearch_global 5_2_COI_assembled_count.fq -db COI_all_OTUs_2020-06-09.txt -strand both -id 0.97 -maxhits 1 -blast6out 5_2_COI_OTUs.txt

/home/f6nv175/usearch11.0.667 -usearch_global 5_3_COI_assembled_count.fq -db COI_all_OTUs_2020-06-09.txt -strand both -id 0.97 -maxhits 1 -blast6out 5_3_COI_OTUs.txt

/home/f6nv175/usearch11.0.667 -usearch_global 6_1a_COI_assembled_count.fq -db COI_all_OTUs_2020-06-09.txt -strand both -id 0.97 -maxhits 1 -blast6out 6_1a_COI_OTUs.txt

/home/f6nv175/usearch11.0.667 -usearch_global 6_1b_2020-06-08_COI_extracted_assembled_count.fq -db COI_all_OTUs_2020-06-09.txt -strand both -id 0.97 -maxhits 1 -blast6out 6_1b_2020-06-09_COI_extracted_OTUs.txt

/home/f6nv175/usearch11.0.667 -usearch_global 6_2_2020-06-08_COI_extracted_assembled_count.fq -db COI_all_OTUs_2020-06-09.txt -strand both -id 0.97 -maxhits 1 -blast6out 6_2_2020-06-09_COI_extracted_OTUs.txt

/home/f6nv175/usearch11.0.667 -usearch_global 6_3_COI_assembled_count.fq -db COI_all_OTUs_2020-06-09.txt -strand both -id 0.97 -maxhits 1 -blast6out 6_3_COI_OTUs.txt

/home/f6nv175/usearch11.0.667 -usearch_global H2O-90A__COI_assembled_count.fq -db COI_all_OTUs_2020-06-09.txt -strand both -id 0.97 -maxhits 1 -blast6out H2O-90A__COI_OTUs.txt

/home/f6nv175/usearch11.0.667 -usearch_global H2O90-B__COI_assembled_count.fq -db COI_all_OTUs_2020-06-09.txt -strand both -id 0.97 -maxhits 1 -blast6out H2O90-B__COI_OTUs.txt

/home/f6nv175/usearch11.0.667 -usearch_global nega_ctrl_1_COI_assembled_count.fq -db COI_all_OTUs_2020-06-09.txt -strand both -id 0.97 -maxhits 1 -blast6out nega_ctrl_1_COI_OTUs.txt

/home/f6nv175/usearch11.0.667 -usearch_global neg_ctrl_3_COI_assembled_count.fq -db COI_all_OTUs_2020-06-09.txt -strand both -id 0.97 -maxhits 1 -blast6out neg_ctrl_3_COI_OTUs.txt

/home/f6nv175/usearch11.0.667 -usearch_global neg_ctrll_2_COI_assembled_count.fq -db COI_all_OTUs_2020-06-09.txt -strand both -id 0.97 -maxhits 1 -blast6out neg_ctrll_2_COI_OTUs.txt

## **7) BLAST**

blastn -db /n/holyscratch01/external_repos/INFORMATICS/ref/ncbi/nt/20200203/nt -num_threads 16 -max_target_seqs 5 -outfmt '6 qseqid sseqid evalue pident sacc stitle' -query COI_all_OTUs_2020-06-09.txt -out COI_all_OTUs_BLAST_nt_2020-06-10

## **8) Add taxonomy to Blast hits**

#remove BLAST hists with non standard NCBI numbers

awk 'length($5)<10' COI_all_OTUs_BLAST_nt_2020-06-10 > COI_all_OTUs_BLAST_nt_out_no_NCBI_2020-06-15

#COI BLAST Table includes 5 hits per OTU, to remove duplicates and keep only first hit per OTU:

awk '!seen[$1]++' COI_all_OTUs_BLAST_nt_out_no_NCBI_2020-06-15 > COI_all_OTUs_BLAST_nt_out_noDup_2020-06-15

# **reduce nucl_gb.accession2taxid to include only matching entries**

awk 'NR==FNR{inFileA[$5]; next} ($1 in inFileA)' COI_all_OTUs_BLAST_nt_out_noDup_2020-06-15 ../nucl_gb.accession2taxid_2020-06-25 > COI_nt_nucl_gb.accession2taxid_2020-07-06

#to add TaxID of NCBI to OTU table:

awk -v OFS='\t' 'NR==FNR{a[$1]=$3; next} ($5 in a) {print $0, a[$5]}' COI_nt_nucl_gb.accession2taxid_2020-07-06 COI_all_OTUs_BLAST_nt_out_noDup_2020-06-15 > COI_all_OTUs_BLAST_nt_out_TaxID_2020-07-06

## **9) Retrieve taxonomy and species information**

curl -s "https://eutils.ncbi.nlm.nih.gov/entrez/eutils/efetch.fcgi?db=taxonomy&amp;id=127563&amp;retmode=xml&amp;rettype=full" | xmllint --xpath "/TaxaSet/Taxon/Lineage/text()" - >> OTU_1_taxonomy_2020-06-16

- the number highlighted in red is taxid; this gives the full taxonomy for this tax id! (to the next HIGHEST RANK; e.g., if species taxid is given it proves all to genus level

curl -s "https://eutils.ncbi.nlm.nih.gov/entrez/eutils/efetch.fcgi?db=taxonomy&amp;id=127563&amp;retmode=xml&amp;rettype=full" | xmllint --xpath "/TaxaSet/Taxon/ScientificName/text()" - >> OTU_1_species_name_2020-06-16

- the number highlighted in red is taxid; this gives the species name
- this creates two separate file for each OTU, one containing the taxonomy and the species name
- these need to concatenated into single files:

sed -e '$s/$//' -s OTU*name_2020-* > COI_nt_all_OTUs_species_names_2020-07-06

sed -e '$s/$//' -s OTU*taxonomy_2020-* > COI_nt_all_OTUs_full_taxonomy_2020-07-06

sort COI_all_OTUs_BLAST_nt_out_TaxID_2020-07-06 -k1 > COI_all_OTUs_BLAST_nt_out_TaxID_sorted_2020-07-06

- COI_all_OTUs_BLAST_nt_out_TaxID_sorted_2020-07-06 COI_nt_all_OTUs_full_taxonomy_2020-07-06 and COI_nt_all_OTUs_species_names_2020-07-06 were merged in Excel
- final file with all information: COI_all_OTUs_BLAST_nt_taxonomy_2020-07-06

## **10) sum OTU frequency in each sample**

To add taxon information to the sample specific OTU files, unique OTUs need to be collapsed and their frequency summed up; done in two steps, first using “cut” to remove remnants of Illumina headers; secondly remove duplicates and add the sum using awk; in the final file there will be two columns 1. OTU name, 2. its frequency in the respective sample

cut -sd'=' -f2 1_1_COI_OTUs.txt > 1_1_COI_OTUs_shortened_2020-06-16.txt

cut -sd'=' -f2 1_2_COI_OTUs.txt > 1_2_COI_OTUs_shortened_2020-06-16.txt

cut -sd'=' -f2 1_3_COI_OTUs.txt > 1_3_COI_OTUs_shortened_2020-06-16.txt

cut -sd'=' -f2 1_4_COI_OTUs.txt > 1_4_COI_OTUs_shortened_2020-06-16.txt

cut -sd'=' -f2 1_5_COI_OTUs.txt > 1_5_COI_OTUs_shortened_2020-06-16.txt

cut -sd'=' -f2 1_6_COI_OTUs.txt > 1_6_COI_OTUs_shortened_2020-06-16.txt

cut -sd'=' -f2 2_1a_COI_OTUs.txt > 2_1a_COI_OTUs_shortened_2020-06-16.txt

cut -sd'=' -f2 2_1b_COI_OTUs.txt > 2_1b_COI_OTUs_shortened_2020-06-16.txt

cut -sd'=' -f2 2_2_COI_OTUs.txt > 2_2_COI_OTUs_shortened_2020-06-16.txt

cut -sd'=' -f2 2_3_COI_OTUs.txt > 2_3_COI_OTUs_shortened_2020-06-16.txt

cut -sd'=' -f2 3_1_COI_OTUs.txt > 3_1_COI_OTUs_shortened_2020-06-16.txt

cut -sd'=' -f2 3_2a_COI_OTUs.txt > 3_2a_COI_OTUs_shortened_2020-06-16.txt

cut -sd'=' -f2 3_2b_COI_OTUs.txt > 3_2b_COI_OTUs_shortened_2020-06-16.txt

cut -sd'=' -f2 3_3_COI_OTUs.txt > 3_3_COI_OTUs_shortened_2020-06-16.txt

cut -sd'=' -f2 4_1_COI_OTUs.txt > 4_1_COI_OTUs_shortened_2020-06-16.txt

cut -sd'=' -f2 4_2_COI_OTUs.txt > 4_2_COI_OTUs_shortened_2020-06-16.txt

cut -sd'=' -f2 4_3_COI_OTUs.txt > 4_3_COI_OTUs_shortened_2020-06-16.txt

cut -sd'=' -f2 4_4_COI_OTUs.txt > 4_4_COI_OTUs_shortened_2020-06-16.txt

cut -sd'=' -f2 4_5_COI_OTUs.txt > 4_5_COI_OTUs_shortened_2020-06-16.txt

cut -sd'=' -f2 4_6_COI_OTUs.txt > 4_6_COI_OTUs_shortened_2020-06-16.txt

cut -sd'=' -f2 5_1_COI_OTUs.txt > 5_1_COI_OTUs_shortened_2020-06-16.txt

cut -sd'=' -f2 5_2_COI_OTUs.txt > 5_2_COI_OTUs_shortened_2020-06-16.txt

cut -sd'=' -f2 5_3_COI_OTUs.txt > 5_3_COI_OTUs_shortened_2020-06-16.txt

cut -sd'=' -f2 6_1a_COI_OTUs.txt > 6_1a_COI_OTUs_shortened_2020-06-16.txt

cut -sd'=' -f2 6_3_COI_OTUs.txt > 6_3_COI_OTUs_shortened_2020-06-16.txt

cut -sd'=' -f2 H2O-90A__COI_OTUs.txt > H2O-90A__COI_OTUs_shortened_2020-06-16.txt

cut -sd'=' -f2 H2O90-B__COI_OTUs.txt > H2O90-B__COI_OTUs_shortened_2020-06-16.txt

cut -sd'=' -f2 nega_ctrl_1_COI_OTUs.txt > nega_ctrl_1_COI_OTUs_shortened_2020-06-16.txt

cut -sd'=' -f2 neg_ctrl_3_COI_OTUs.txt > neg_ctrl_3_COI_OTUs_shortened_2020-06-16.txt

cut -sd'=' -f2 neg_ctrll_2_COI_OTUs.txt > neg_ctrll_2_COI_OTUs_shortened_2020-06-16.txt

cut -sd'=' -f2 6_1b_2020-06-09_COI_extracted_OTUs.txt > 6_1b_COI_OTUs_shortened_2020-06-16.txt

cut -sd'=' -f2 6_2_2020-06-09_COI_extracted_OTUs.txt > 6_2_COI_OTUs_shortened_2020-06-16.txt

awk -v OFS='\t' '{ seen[$2] += $1 } END { for (i in seen) print i, seen[i] }' 1_1_COI_OTUs_shortened_2020-06-16.txt > 1_1_COI_OTUs_shortened_OTU_collapsed_2020-06-16.txt

awk -v OFS='\t' '{ seen[$2] += $1 } END { for (i in seen) print i, seen[i] }' 1_2_COI_OTUs_shortened_2020-06-16.txt > 1_2_COI_OTUs_shortened_OTU_collapsed_2020-06-16.txt

awk -v OFS='\t' '{ seen[$2] += $1 } END { for (i in seen) print i, seen[i] }' 1_3_COI_OTUs_shortened_2020-06-16.txt > 1_3_COI_OTUs_shortened_OTU_collapsed_2020-06-16.txt

awk -v OFS='\t' '{ seen[$2] += $1 } END { for (i in seen) print i, seen[i] }' 1_4_COI_OTUs_shortened_2020-06-16.txt > 1_4_COI_OTUs_shortened_OTU_collapsed_2020-06-16.txt

awk -v OFS='\t' '{ seen[$2] += $1 } END { for (i in seen) print i, seen[i] }' 1_5_COI_OTUs_shortened_2020-06-16.txt > 1_5_COI_OTUs_shortened_OTU_collapsed_2020-06-16.txt

awk -v OFS='\t' '{ seen[$2] += $1 } END { for (i in seen) print i, seen[i] }' 1_6_COI_OTUs_shortened_2020-06-16.txt > 1_6_COI_OTUs_shortened_OTU_collapsed_2020-06-16.txt

awk -v OFS='\t' '{ seen[$2] += $1 } END { for (i in seen) print i, seen[i] }' 2_1a_COI_OTUs_shortened_2020-06-16.txt > 2_1a_COI_OTUs_shortened_OTU_collapsed_2020-06-16.txt

awk -v OFS='\t' '{ seen[$2] += $1 } END { for (i in seen) print i, seen[i] }' 2_1b_COI_OTUs_shortened_2020-06-16.txt > 2_1b_COI_OTUs_shortened_OTU_collapsed_2020-06-16.txt

awk -v OFS='\t' '{ seen[$2] += $1 } END { for (i in seen) print i, seen[i] }' 2_2_COI_OTUs_shortened_2020-06-16.txt > 2_2_COI_OTUs_shortened_OTU_collapsed_2020-06-16.txt

awk -v OFS='\t' '{ seen[$2] += $1 } END { for (i in seen) print i, seen[i] }' 2_3_COI_OTUs_shortened_2020-06-16.txt > 2_3_COI_OTUs_shortened_OTU_collapsed_2020-06-16.txt

awk -v OFS='\t' '{ seen[$2] += $1 } END { for (i in seen) print i, seen[i] }' 3_1_COI_OTUs_shortened_2020-06-16.txt > 3_1_COI_OTUs_shortened_OTU_collapsed_2020-06-16.txt

awk -v OFS='\t' '{ seen[$2] += $1 } END { for (i in seen) print i, seen[i] }' 3_2a_COI_OTUs_shortened_2020-06-16.txt > 3_2a_COI_OTUs_shortened_OTU_collapsed_2020-06-16.txt

awk -v OFS='\t' '{ seen[$2] += $1 } END { for (i in seen) print i, seen[i] }' 3_2b_COI_OTUs_shortened_2020-06-16.txt > 3_2b_COI_OTUs_shortened_OTU_collapsed_2020-06-16.txt

awk -v OFS='\t' '{ seen[$2] += $1 } END { for (i in seen) print i, seen[i] }' 3_3_COI_OTUs_shortened_2020-06-16.txt > 3_3_COI_OTUs_shortened_OTU_collapsed_2020-06-16.txt

awk -v OFS='\t' '{ seen[$2] += $1 } END { for (i in seen) print i, seen[i] }' 4_2_COI_OTUs_shortened_2020-06-16.txt > 4_2_COI_OTUs_shortened_OTU_collapsed_2020-06-16.txt

awk -v OFS='\t' '{ seen[$2] += $1 } END { for (i in seen) print i, seen[i] }' 4_3_COI_OTUs_shortened_2020-06-16.txt > 4_3_COI_OTUs_shortened_OTU_collapsed_2020-06-16.txt

awk -v OFS='\t' '{ seen[$2] += $1 } END { for (i in seen) print i, seen[i] }' 4_4_COI_OTUs_shortened_2020-06-16.txt > 4_4_COI_OTUs_shortened_OTU_collapsed_2020-06-16.txt

awk -v OFS='\t' '{ seen[$2] += $1 } END { for (i in seen) print i, seen[i] }' 4_5_COI_OTUs_shortened_2020-06-16.txt > 4_5_COI_OTUs_shortened_OTU_collapsed_2020-06-16.txt

awk -v OFS='\t' '{ seen[$2] += $1 } END { for (i in seen) print i, seen[i] }' 4_6_COI_OTUs_shortened_2020-06-16.txt > 4_6_COI_OTUs_shortened_OTU_collapsed_2020-06-16.txt

awk -v OFS='\t' '{ seen[$2] += $1 } END { for (i in seen) print i, seen[i] }' 5_1_COI_OTUs_shortened_2020-06-16.txt > 5_1_COI_OTUs_shortened_OTU_collapsed_2020-06-16.txt

awk -v OFS='\t' '{ seen[$2] += $1 } END { for (i in seen) print i, seen[i] }' 5_2_COI_OTUs_shortened_2020-06-16.txt > 5_2_COI_OTUs_shortened_OTU_collapsed_2020-06-16.txt

awk -v OFS='\t' '{ seen[$2] += $1 } END { for (i in seen) print i, seen[i] }' 5_3_COI_OTUs_shortened_2020-06-16.txt > 5_3_COI_OTUs_shortened_OTU_collapsed_2020-06-16.txt

awk -v OFS='\t' '{ seen[$2] += $1 } END { for (i in seen) print i, seen[i] }' 6_1a_COI_OTUs_shortened_2020-06-16.txt > 6_1a_COI_OTUs_shortened_OTU_collapsed_2020-06-16.txt

awk -v OFS='\t' '{ seen[$2] += $1 } END { for (i in seen) print i, seen[i] }' 6_3_COI_OTUs_shortened_2020-06-16.txt > 6_3_COI_OTUs_shortened_OTU_collapsed_2020-06-16.txt

awk -v OFS='\t' '{ seen[$2] += $1 } END { for (i in seen) print i, seen[i] }' H2O-90A__COI_OTUs_shortened_2020-06-16.txt > H2O-90A__COI_OTUs_shortened_OTU_collapsed_2020-06-16.txt

awk -v OFS='\t' '{ seen[$2] += $1 } END { for (i in seen) print i, seen[i] }' H2O90-B__COI_OTUs_shortened_2020-06-16.txt > H2O90-B__COI_OTUs_shortened_OTU_collapsed_2020-06-16.txt

awk -v OFS='\t' '{ seen[$2] += $1 } END { for (i in seen) print i, seen[i] }' nega_ctrl_1_COI_OTUs_shortened_2020-06-16.txt > nega_ctrl_1_COI_OTUs_shortened_OTU_collapsed_2020-06-16.txt

awk -v OFS='\t' '{ seen[$2] += $1 } END { for (i in seen) print i, seen[i] }' neg_ctrl_3_COI_OTUs_shortened_2020-06-16.txt > neg_ctrl_3_COI_OTUs_shortened_OTU_collapsed_2020-06-16.txt

awk -v OFS='\t' '{ seen[$2] += $1 } END { for (i in seen) print i, seen[i] }' neg_ctrll_2_COI_OTUs_shortened_2020-06-16.txt > neg_ctrll_2_COI_OTUs_shortened_OTU_collapsed_2020-06-16.txt

awk -v OFS='\t' '{ seen[$2] += $1 } END { for (i in seen) print i, seen[i] }' 4_1_COI_OTUs_shortened_2020-06-16.txt > 4_1_COI_OTUs_shortened_OTU_collapsed_2020-06-16.txt

awk -v OFS='\t' '{ seen[$2] += $1 } END { for (i in seen) print i, seen[i] }' 6_1b_COI_OTUs_shortened_2020-06-16.txt > 6_1b_COI_OTUs_shortened_OTU_collapsed_2020-06-16.txt

awk -v OFS='\t' '{ seen[$2] += $1 } END { for (i in seen) print i, seen[i] }' 6_2_COI_OTUs_shortened_2020-06-16.txt > 6_2_COI_OTUs_shortened_OTU_collapsed_2020-06-16.txt

## **11) add taxonomy information to each sample**

This adds the taxonomy info to each OTU retrieved in each single sample.

**ATTENTION: OTUs without BLAST HIT will be REMOVED from the output**

**the suitable header for the resulting files is:**

**OTU_number OTU_count/frequency OTU_number GB_number e-value percentage NCBI info TaxID Taxonomy** Species

awk -v OFS='\t' 'NR==FNR{a[$1]=$0; next} ($1 in a) {print $0, a[$1]}' COI_all_OTUs_BLAST_nt_taxonomy_2020-07-06 1_1_COI_OTUs_shortened_OTU_collapsed_2020-06-16.txt > 1_1_COI_OTUs_shortened_OTU_collapsed_taxonomy_2020-07-06.txt

awk -v OFS='\t' 'NR==FNR{a[$1]=$0; next} ($1 in a) {print $0, a[$1]}' COI_all_OTUs_BLAST_nt_taxonomy_2020-07-06 1_2_COI_OTUs_shortened_OTU_collapsed_2020-06-16.txt > 1_2_COI_OTUs_shortened_OTU_collapsed_taxonomy_2020-07-06.txt

awk -v OFS='\t' 'NR==FNR{a[$1]=$0; next} ($1 in a) {print $0, a[$1]}' COI_all_OTUs_BLAST_nt_taxonomy_2020-07-06 1_3_COI_OTUs_shortened_OTU_collapsed_2020-06-16.txt > 1_3_COI_OTUs_shortened_OTU_collapsed_taxonomy_2020-07-06.txt

awk -v OFS='\t' 'NR==FNR{a[$1]=$0; next} ($1 in a) {print $0, a[$1]}' COI_all_OTUs_BLAST_nt_taxonomy_2020-07-06 1_4_COI_OTUs_shortened_OTU_collapsed_2020-06-16.txt > 1_4_COI_OTUs_shortened_OTU_collapsed_taxonomy_2020-07-06.txt

awk -v OFS='\t' 'NR==FNR{a[$1]=$0; next} ($1 in a) {print $0, a[$1]}' COI_all_OTUs_BLAST_nt_taxonomy_2020-07-06 1_5_COI_OTUs_shortened_OTU_collapsed_2020-06-16.txt > 1_5_COI_OTUs_shortened_OTU_collapsed_taxonomy_2020-07-06.txt

awk -v OFS='\t' 'NR==FNR{a[$1]=$0; next} ($1 in a) {print $0, a[$1]}' COI_all_OTUs_BLAST_nt_taxonomy_2020-07-06 1_6_COI_OTUs_shortened_OTU_collapsed_2020-06-16.txt > 1_6_COI_OTUs_shortened_OTU_collapsed_taxonomy_2020-07-06.txt

awk -v OFS='\t' 'NR==FNR{a[$1]=$0; next} ($1 in a) {print $0, a[$1]}' COI_all_OTUs_BLAST_nt_taxonomy_2020-07-06 2_1a_COI_OTUs_shortened_OTU_collapsed_2020-06-16.txt > 2_1a_COI_OTUs_shortened_OTU_collapsed_taxonomy_2020-07-06.txt

awk -v OFS='\t' 'NR==FNR{a[$1]=$0; next} ($1 in a) {print $0, a[$1]}' COI_all_OTUs_BLAST_nt_taxonomy_2020-07-06 2_1b_COI_OTUs_shortened_OTU_collapsed_2020-06-16.txt > 2_1b_COI_OTUs_shortened_OTU_collapsed_taxonomy_2020-07-06.txt

awk -v OFS='\t' 'NR==FNR{a[$1]=$0; next} ($1 in a) {print $0, a[$1]}' COI_all_OTUs_BLAST_nt_taxonomy_2020-07-06 2_2_COI_OTUs_shortened_OTU_collapsed_2020-06-16.txt > 2_2_COI_OTUs_shortened_OTU_collapsed_taxonomy_2020-07-06.txt

awk -v OFS='\t' 'NR==FNR{a[$1]=$0; next} ($1 in a) {print $0, a[$1]}' COI_all_OTUs_BLAST_nt_taxonomy_2020-07-06 2_3_COI_OTUs_shortened_OTU_collapsed_2020-06-16.txt > 2_3_COI_OTUs_shortened_OTU_collapsed_taxonomy_2020-07-06.txt

awk -v OFS='\t' 'NR==FNR{a[$1]=$0; next} ($1 in a) {print $0, a[$1]}' COI_all_OTUs_BLAST_nt_taxonomy_2020-07-06 3_1_COI_OTUs_shortened_OTU_collapsed_2020-06-16.txt > 3_1_COI_OTUs_shortened_OTU_collapsed_taxonomy_2020-07-06.txt

awk -v OFS='\t' 'NR==FNR{a[$1]=$0; next} ($1 in a) {print $0, a[$1]}' COI_all_OTUs_BLAST_nt_taxonomy_2020-07-06 3_2a_COI_OTUs_shortened_OTU_collapsed_2020-06-16.txt > 3_2a_COI_OTUs_shortened_OTU_collapsed_taxonomy_2020-07-06.txt

awk -v OFS='\t' 'NR==FNR{a[$1]=$0; next} ($1 in a) {print $0, a[$1]}' COI_all_OTUs_BLAST_nt_taxonomy_2020-07-06 3_2b_COI_OTUs_shortened_OTU_collapsed_2020-06-16.txt > 3_2b_COI_OTUs_shortened_OTU_collapsed_taxonomy_2020-07-06.txt

awk -v OFS='\t' 'NR==FNR{a[$1]=$0; next} ($1 in a) {print $0, a[$1]}' COI_all_OTUs_BLAST_nt_taxonomy_2020-07-06 3_3_COI_OTUs_shortened_OTU_collapsed_2020-06-16.txt > 3_3_COI_OTUs_shortened_OTU_collapsed_taxonomy_2020-07-06.txt

awk -v OFS='\t' 'NR==FNR{a[$1]=$0; next} ($1 in a) {print $0, a[$1]}' COI_all_OTUs_BLAST_nt_taxonomy_2020-07-06 4_2_COI_OTUs_shortened_OTU_collapsed_2020-06-16.txt > 4_2_COI_OTUs_shortened_OTU_collapsed_taxonomy_2020-07-06.txt

awk -v OFS='\t' 'NR==FNR{a[$1]=$0; next} ($1 in a) {print $0, a[$1]}' COI_all_OTUs_BLAST_nt_taxonomy_2020-07-06 4_3_COI_OTUs_shortened_OTU_collapsed_2020-06-16.txt > 4_3_COI_OTUs_shortened_OTU_collapsed_taxonomy_2020-07-06.txt

awk -v OFS='\t' 'NR==FNR{a[$1]=$0; next} ($1 in a) {print $0, a[$1]}' COI_all_OTUs_BLAST_nt_taxonomy_2020-07-06 4_4_COI_OTUs_shortened_OTU_collapsed_2020-06-16.txt > 4_4_COI_OTUs_shortened_OTU_collapsed_taxonomy_2020-07-06.txt

awk -v OFS='\t' 'NR==FNR{a[$1]=$0; next} ($1 in a) {print $0, a[$1]}' COI_all_OTUs_BLAST_nt_taxonomy_2020-07-06 4_5_COI_OTUs_shortened_OTU_collapsed_2020-06-16.txt > 4_5_COI_OTUs_shortened_OTU_collapsed_taxonomy_2020-07-06.txt

awk -v OFS='\t' 'NR==FNR{a[$1]=$0; next} ($1 in a) {print $0, a[$1]}' COI_all_OTUs_BLAST_nt_taxonomy_2020-07-06 4_6_COI_OTUs_shortened_OTU_collapsed_2020-06-16.txt > 4_6_COI_OTUs_shortened_OTU_collapsed_taxonomy_2020-07-06.txt

awk -v OFS='\t' 'NR==FNR{a[$1]=$0; next} ($1 in a) {print $0, a[$1]}' COI_all_OTUs_BLAST_nt_taxonomy_2020-07-06 5_1_COI_OTUs_shortened_OTU_collapsed_2020-06-16.txt > 5_1_COI_OTUs_shortened_OTU_collapsed_taxonomy_2020-07-06.txt

awk -v OFS='\t' 'NR==FNR{a[$1]=$0; next} ($1 in a) {print $0, a[$1]}' COI_all_OTUs_BLAST_nt_taxonomy_2020-07-06 5_2_COI_OTUs_shortened_OTU_collapsed_2020-06-16.txt > 5_2_COI_OTUs_shortened_OTU_collapsed_taxonomy_2020-07-06.txt

awk -v OFS='\t' 'NR==FNR{a[$1]=$0; next} ($1 in a) {print $0, a[$1]}' COI_all_OTUs_BLAST_nt_taxonomy_2020-07-06 5_3_COI_OTUs_shortened_OTU_collapsed_2020-06-16.txt > 5_3_COI_OTUs_shortened_OTU_collapsed_taxonomy_2020-07-06.txt

awk -v OFS='\t' 'NR==FNR{a[$1]=$0; next} ($1 in a) {print $0, a[$1]}' COI_all_OTUs_BLAST_nt_taxonomy_2020-07-06 6_1a_COI_OTUs_shortened_OTU_collapsed_2020-06-16.txt > 6_1a_COI_OTUs_shortened_OTU_collapsed_taxonomy_2020-07-06.txt

awk -v OFS='\t' 'NR==FNR{a[$1]=$0; next} ($1 in a) {print $0, a[$1]}' COI_all_OTUs_BLAST_nt_taxonomy_2020-07-06 6_3_COI_OTUs_shortened_OTU_collapsed_2020-06-16.txt > 6_3_COI_OTUs_shortened_OTU_collapsed_taxonomy_2020-07-06.txt

awk -v OFS='\t' 'NR==FNR{a[$1]=$0; next} ($1 in a) {print $0, a[$1]}' COI_all_OTUs_BLAST_nt_taxonomy_2020-07-06 H2O-90A__COI_OTUs_shortened_OTU_collapsed_2020-06-16.txt > H2O-90A__COI_OTUs_shortened_OTU_collapsed_taxonomy_2020-07-06.txt

awk -v OFS='\t' 'NR==FNR{a[$1]=$0; next} ($1 in a) {print $0, a[$1]}' COI_all_OTUs_BLAST_nt_taxonomy_2020-07-06 H2O90-B__COI_OTUs_shortened_OTU_collapsed_2020-06-16.txt > H2O90-B__COI_OTUs_shortened_OTU_collapsed_taxonomy_2020-07-06.txt

awk -v OFS='\t' 'NR==FNR{a[$1]=$0; next} ($1 in a) {print $0, a[$1]}' COI_all_OTUs_BLAST_nt_taxonomy_2020-07-06 nega_ctrl_1_COI_OTUs_shortened_OTU_collapsed_2020-06-16.txt > nega_ctrl_1_COI_OTUs_shortened_OTU_collapsed_taxonomy_2020-07-06.txt

awk -v OFS='\t' 'NR==FNR{a[$1]=$0; next} ($1 in a) {print $0, a[$1]}' COI_all_OTUs_BLAST_nt_taxonomy_2020-07-06 neg_ctrl_3_COI_OTUs_shortened_OTU_collapsed_2020-06-16.txt > neg_ctrl_3_COI_OTUs_shortened_OTU_collapsed_taxonomy_2020-07-06.txt

awk -v OFS='\t' 'NR==FNR{a[$1]=$0; next} ($1 in a) {print $0, a[$1]}' COI_all_OTUs_BLAST_nt_taxonomy_2020-07-06 neg_ctrll_2_COI_OTUs_shortened_OTU_collapsed_2020-06-16.txt > neg_ctrll_2_COI_OTUs_shortened_OTU_collapsed_taxonomy_2020-07-06.txt

awk -v OFS='\t' 'NR==FNR{a[$1]=$0; next} ($1 in a) {print $0, a[$1]}' COI_all_OTUs_BLAST_nt_taxonomy_2020-07-06 4_1_COI_OTUs_shortened_OTU_collapsed_2020-06-16.txt > 4_1_COI_OTUs_shortened_OTU_collapsed_taxonomy_2020-07-06.txt

awk -v OFS='\t' 'NR==FNR{a[$1]=$0; next} ($1 in a) {print $0, a[$1]}' COI_all_OTUs_BLAST_nt_taxonomy_2020-07-06 6_1b_COI_OTUs_shortened_OTU_collapsed_2020-06-16.txt > 6_1b_COI_OTUs_shortened_OTU_collapsed_taxonomy_2020-07-06.txt

awk -v OFS='\t' 'NR==FNR{a[$1]=$0; next} ($1 in a) {print $0, a[$1]}' COI_all_OTUs_BLAST_nt_taxonomy_2020-07-06 6_2_COI_OTUs_shortened_OTU_collapsed_2020-06-16.txt > 6_2_COI_OTUs_shortened_OTU_collapsed_taxonomy_2020-07-06.txt

## **12) create a file that contains all OTUs from all replicates, with original file name added to each line**

awk -v OFS='\t' '{print FILENAME, $0}' *COI_OTUs_shortened_OTU_collapsed_taxonomy_2020-07-06.txt >> COI_all_replicates_taxonomy_2020-07-06

## **13) assign and remove species**

done in Excel

- only Metazoa retained
- based on % similarity:

<85% similarity : unkown Metazoa (same for samples classified as Invertebrate environmental sample)

85-97% : higher classification (fish, crustacean etc) retained, but not species

>97% species identification retained

- based on evalue:

>e-20 : unkown Metazoa

e-20 - e-50: higher classification (fish, crustacean etc) retained, but not species

<e-50 species identification retained

(Both threshold applied together; e.g., to retain species names >97% similarity AND evalue < e-50)

- remove all 12S contaminants
